# Genome-wide transcriptional responses of *Alteromonas naphthalenivorans* SN2 to contaminated seawater and marine tidal flat sediment

**DOI:** 10.1038/srep21796

**Published:** 2016-02-18

**Authors:** Hyun Mi Jin, Hye Im Jeong, Kyung Hyun Kim, Yoonsoo Hahn, Eugene L. Madsen, Che Ok Jeon

**Affiliations:** 1 Department of Life Science, Chung-Ang University, Seoul 06974, Republic of Korea; 2 Freshwater Bioresources Utilization Division, Nakdonggang National Institute of Biological Resources, Gyeongsangbuk-do 37242, Republic of Korea; 3 Department of Microbiology, Cornell University, Ithaca, NY 14853-8101, USA

## Abstract

A genome-wide transcriptional analysis of *Alteromonas naphthalenivorans* SN2 was performed to investigate its ecophysiological behavior in contaminated tidal flats and seawater. The experimental design mimicked these habitats that either added naphthalene or pyruvate; tidal flat-naphthalene (TF-N), tidal flat-pyruvate (TF-P), seawater-naphthalene (SW-N), and seawater-pyruvate (SW-P). The transcriptional profiles clustered by habitat (TF-N/TF-P and SW-N/SW-P), rather than carbon source, suggesting that the former may exert a greater influence on genome-wide expression in strain SN2 than the latter. Metabolic mapping of cDNA reads from strain SN2 based on KEGG pathway showed that metabolic and regulatory genes associated with energy metabolism, translation, and cell motility were highly expressed in all four test conditions, probably highlighting the copiotrophic properties of strain SN2 as an opportunistic marine *r*-strategist. Differential gene expression analysis revealed that strain SN2 displayed specific cellular responses to environmental variables (tidal flat, seawater, naphthalene, and pyruvate) and exhibited certain ecological fitness traits –– its notable PAH degradation capability in seasonally cold tidal flat might be reflected in elevated expression of stress response and chaperone proteins, while fast growth in nitrogen-deficient and aerobic seawater probably correlated with high expression of glutamine synthetase, enzymes utilizing nitrite/nitrate, and those involved in the removal of reactive oxygen species.

Various ecotypes of the genus *Alteromonas*, consisting of Gram-negative, aerobic, moderately halophilic, rod-shaped chemoheterotrophs with polar flagellum motility, have been isolated from marine environments around the world, including surface, deep, and Antarctic seawater, and coastal tidal flats^1^,^2^,^3^,^4^,^5^,^6^,^7^,^8^. Currently, the genus contains 14 species with validly published names (http://www.bacterio.net), most of which, including *Alteromonas macleodii* (the type species of this genus), have been isolated from oceans located in temperate or tropical latitudes and thus show mesophilic growth, i.e. growth between 10 and 45 °C^9^. Just two *Alteromonas* species among these 14 have been isolated from habitats featuring cold temperatures, namely *A. stellipolaris* (Antarctic seawater) and *A. litorea* (tidal-flat sediments with cold winters), respectively^3^,^4^.

Recent studies including metagenomic and metatranscriptomic analyses have shown that members of the order *Alteromonadales* account for the majority of the increase in cell abundance and transcriptional activity in environmental microcosms supplemented with by dissolved organic carbon^10^,^11^,^12^,^13^. Tada *et al.*^14^ reported that the abundance of *Alteromonas* species increased to up to 30% of the total growing bacterial population when a phytoplankton bloom occurred, suggesting that they may play important roles in marine carbon cycles. Previous studies have also shown that *Alteromonas* species display typical copiotrophic physiological capabilities, and thus are generally described as opportunistic marine *r*-strategists^11^,^13^,^15^. Many investigations have demonstrated that some members of this genus are likely to be responsible for hydrocarbon biodegradation in marine environments contaminated by crude oil spills^16^,^17^,^18^. In addition, our own previous work showed that *Alteromonas naphthalenivorans* SN2, isolated from a crude oil-contaminated marine tidal flat in Korea, is a key player in the biodegradation of polycyclic aromatic hydrocarbons (PAHs) in contaminated coastal sediment^19^. Marine tidal flats are coastal muddy areas alternately flooded with seawater and exposed to the atmosphere and strain SN2 grows well and degrades PAH compounds rapidly in both seawater and tidal flat sediment, demonstrating considerable adaptation to both of these environments^6^,^8^,^19^. In addition, this strain has a broad growth temperature range, capable in particular of greater cold tolerance (growing at 5 °C) than other *Alteromonas* species likely due to the characteristics of its habitat, which includes low winter temperatures^6^. Comparative genomic analysis has established that strain SN2 has genomic features distinct from two other *Alteromonas* ecotypes isolated from seawater, AltDE and ATCC 27126^6^,^20^. However, the detailed behavior and adaptive responses of strain SN2 in regard to its environment (including exposure to PAH compounds) have not been explored, although the global reactions of other microbes have been partially studied under mimicking their native habitats^21^,^22^.

The metabolic properties of microorganisms have in the past been elucidated through genomic sequencing analyses that reveal evolutionary selective pressures, but the utility of the DNA-based genomics is limited when attempting to understand fine-scale relationships between ecophysiological adaptations and habitat characteristics. Transcriptomic analysis has been suggested as an approach better suited to the elucidation of such microbial characteristics^23^. Until now, most transcriptomic analyses have been performed in laboratories using artificial media and pure cultures^24^,^25^,^26^,^27^,^28^. However, ecologically-significant metabolic adaptions are far more likely to be discovered under experimental conditions that mimic conditions that occur in the native habitat of the microorganism of interest^21^,^22^.

The objective of this study was to investigate the ecophysiological properties and environmental behavior of *Alteromonas naphthalenivorans* SN2, responsible for PAH biodegradation in a contaminated marine tidal flat. In order to accomplish this, we prepared four environmental conditions mimicking this habitat (tidal flat sediment and seawater amended with naphthalene or pyruvate as carbon substrates) and used an Illumina mRNA-sequencing approach to comprehensively and quantitatively assess genome-wide transcription of strain SN2 under each of these conditions. In particular, we sought to investigate the global and specific responses of strain SN2 to marine tidal flat samples containing naphthalene, compared to those observed in seawater samples containing pyruvate.

## Results and Discussion

### Preparation of TF-N, TF-P, SW-N and SW-P conditions

Tidal flat sediment and seawater samples were collected from a contaminated coastal area approximately six years after an oil spill event. Total PAH concentration in tidal flat sediment was only approximately 10 μg/kg and moreover, PAH compounds were not detected in seawater samples, suggesting that the contaminated area in question had almost recovered. qPCR analysis showed that 16S rRNA gene copy numbers for total bacteria in the tidal flat sediment and seawater samples were approximately 10^10 ^copies/g and 10^5 ^copies/ml, respectively, figures similar to those measured during an earlier period following the oil spill^19^. However, *Alteromonas*-specific 16S rRNA qPCR revealed fewer than 10^4 ^copies/g of tidal flat sediment, representing a decrease of approximately two orders of magnitude compared to the earlier contaminated period^19^. Furthermore, *Alteromonas* 16S rRNA sequences were not detected through qPCR from the seawater samples. These qPCR results lend support to the proposition that copiotrophic *Alteromonas* species dominate heterotrophic blooms when carbon sources are introduced to marine habitats, and play key roles in degrading crude oil components, including PAH compounds^11^,^13^,^15^,^19^,^29^.

To investigate the ecophysiological properties and environmental behavior of *Alteromonas* sp. SN2 in contaminated marine tidal flats and seawater, four environmental test conditions, TF-N, TF-P, SW-N, and SW-P were prepared in the laboratory using tidal flat sediment and seawater samples. With the exception of the TF-P condition, the TF-N, SW-N, and SW-P conditions were prepared using nonsterile tidal flat sediment and seawater (featuring the native, intact microbial communities) to better mimic environmental field conditions. The mimic experimental conditions used serum bottles and were closed systems, while the contaminated field sites are open systems. It would have been impossible to directly perform the desired tests in the field study site (tidal flat). These methodological limitations force us to declare that the model systems used here were imperfect. Necessarily then, some interactions between *Alteromonas* species and other organisms or environmental factors present in an open, tidal-flat field site may have been overlooked. For the tidal flat conditions (TF-N and TF-P), strain SN2 was inoculated into the test mixtures to approximate the cell numbers of *Alteromonas* species (approximately 10^6 ^cells/g of sediment) observed in samples taken from this habitat during the earlier contaminated period^19^. However, for the seawater conditions (SW-N and SW-P), cells of strain SN2 were inoculated to be approximately 10^7 ^cells/ml, as this strain failed to grow in the SW-N test condition using a concentration of 10^6 ^cells/ml, probably due to naphthalene toxicity^30^.

### Growth of strain SN2 and mRNA purification

**Figure 1:**
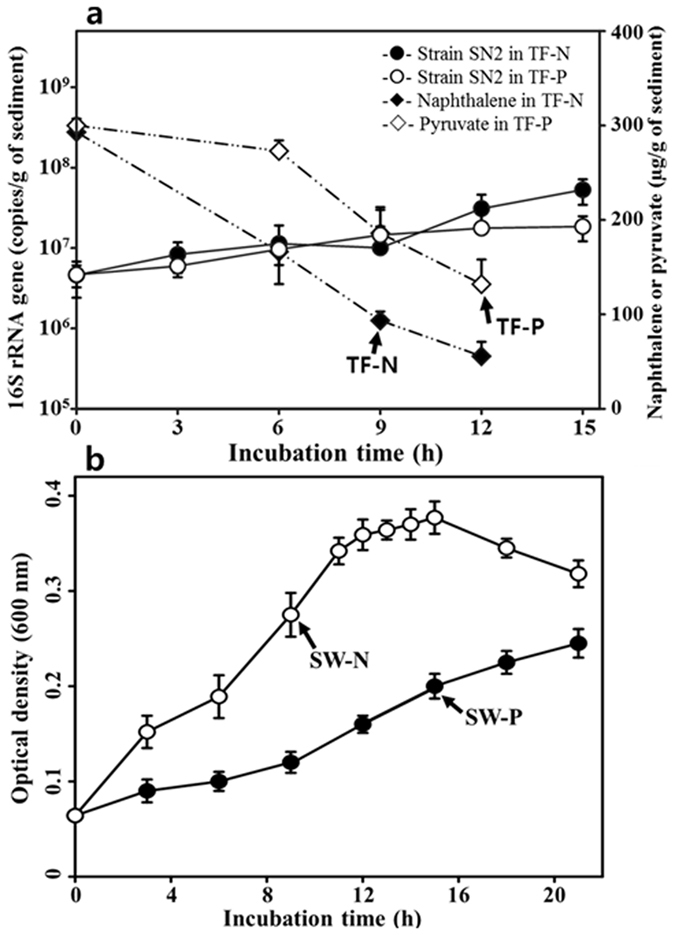
Growth of strain SN2 in tidal flat sediment (**a**) and seawater (**b**) supplemented with naphthalene or pyruvate. Arrows indicate the time at which cells were harvested for mRNA extraction. The number of 16S rRNA gene copies of strain SN2 and the concentrations of naphthalene and pyruvate in tidal flat sediment and the optical density in seawater were measured using three independent cultures.

With a view to extracting mRNA from strain SN2 cells recovered during the exponential growth phase in the four environmental test conditions, cell growth was monitored. In the TF-N and TF-P conditions, the concentrations of naphthalene and pyruvate decreased concomitantly with the increase of *Alteromonas* cell numbers (Fig. 1a). Interestingly, the concentration of naphthalene decreased faster than that of pyruvate, supporting a previous observation that strain SN2 displays a robust ability to biodegrade PAH compounds^19^. When the concentrations of naphthalene and pyruvate reached approximately 100 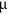
g/g of sediment (after 9 and 12 h following inoculation, respectively), tidal flat slurries were removed from the serum bottles for mRNA extraction. We concluded that a transcriptomic analysis of strain SN2 using total mRNA derived from all bacteria present in the tidal flat slurries would be impossible owing to the fact that the number of strain SN2 cells (approximately 10^6 ^cells/g of sediment) was approximately three to four orders of magnitude lower than the total native bacterial cell count (approximately 10^10 ^cells/g of sediment). Therefore, for the TF-N condition, serum bottles containing ^13^C-naphthalene, rather than those with ^12^C-naphthalene, were used for the mRNA extraction, and transcripts derived from naphthalene degraders (predominantly strain SN2) were enriched using isopycnic ultracentrifugation in CsTFA gradients. RT-qPCR of *gyrB* transcripts obtained from the CsTFA gradient fractions clearly showed that the tidal flat slurries incubated with ^12^C-naphthalene yielded a single peak of ‘light’ mRNA transcripts (data not shown), whereas those incubated with ^13^C-naphthalene included an additional peak (with a density of approximately 1.81 to 1.85 g/ml) containing ‘heavy’ transcripts (Supplementary Fig. 1). However, enrichment of strain SN2 mRNA by isopycnic ultracentrifugation was not applied to the TF-P samples, as the metabolism of pyruvate is not limited to naphthalene degraders; instead and extremely broad diversity of bacteria present in the tidal flat slurries are expected to be capable of this. Therefore, sterile tidal flat sediment had to be used for the TF-P experimental treatment despite the fact that nonsterile sediment likely better represents *in situ* tidal flat characteristics.

Nonsterile seawater was used for both SW-N and SW-P conditions because the number of strain SN2 cells inoculated (approximately 10^7 ^cells/ml; approximately two orders of magnitude higher than the total native cells) allowed recovery of SN2 transcripts. The proliferation of strain SN2 in these test conditions was faster in naphthalene than in pyruvate, as observed for the tidal flat conditions (Fig. 1b).

### Illumina sequencing and bioinformatic data processing

Raw cDNA sequence reads derived from TF-N, TF-P, SW-N, and SW-P conditions were processed by removing low quality (quality score <23) and short length (<50 bases) reads, and more than 10 million clean cDNA reads for each condition were obtained (Table 1). Although it was presumed that the abundance of strain SN2 was around three orders of magnitude lower than that of the total bacterial cell count, 55.3% of the clean cDNA reads derived from the TF-N condition mapped onto the genome of strain SN2, suggesting that the enrichment of this strain’s mRNA using ^13^C-naphthalene and isopycnic centrifugation was effective. On the other hand, although the tidal flat sediment and seawater samples used for the TF-P condition were sterilized, only 35.6% of the clean cDNA reads mapped onto the genome of strain SN2, indicating that a considerable amount of mRNA derived from dead microorganisms was sequenced without mRNA degradation by autoclaving. More than 98.6% of the clean cDNA reads from the seawater conditions successfully mapped onto the genome of strain SN2, which was understandable given the relatively high concentration of strain SN2 cells in the SW-N and SW-P conditions (10^7 ^cells/ml compared to a pre-inoculation total bacterial cell count of 10^5 ^cells/ml). The percentages of clean cDNA reads mapping onto CDSs in the genome of strain SN2 ranged from 21.6 to 98.8% of the total clean cDNA reads, indicating that structural RNAs (16S and 23S rRNA) were efficiently eliminated by the rRNA removal step.

### Genome-wide transcriptional profiles of strain SN2

Frequency distributions of the RPKM values for all CDSs present in the genome of strain SN2 were similar for each of the four environmental test conditions (Supplementary Fig. 2), indicating that the preparation and sequencing of mRNA derived from these experiments were carried out appropriately.

**Figure 2:**
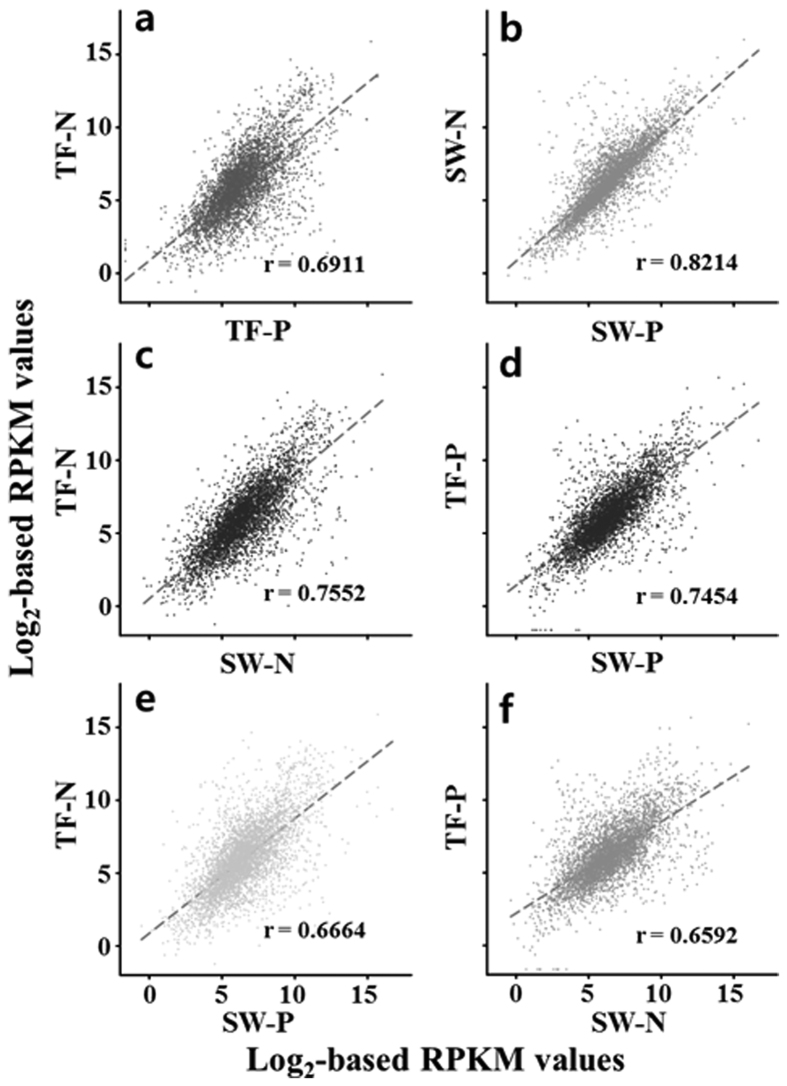
Scatter plot comparisons of the transcriptome of strain SN2 in four environmental test conditions, TF-N, TF-P, SW-N, and SW-P. The r figures indicate correlation coefficients between the RPKM values (read number per kb of each coding sequence, per million mapped sequences) of two representative test conditions. TF, tidal flat; SW, seawater; P, pyruvate; N, naphthalene.

Genome-wide transcriptional profiles of strain SN2 from the environmental test conditions were compared via scatter plotting of the RPKM values. Figure. 2a,b demonstrated that the SW-N and SW-P profiles were considerably more similar to each other than those generated from TF-N and TF-P conditions, suggesting that gene transcription in strain SN2 may be less influenced by carbon sources in seawater than it is in tidal flats. In addition, the resemblance between the transcriptional profiles from TF-N and SW-N conditions was slightly greater than that between the TF-P and SW-P profiles (Fig. 2c,d), which may imply that naphthalene exerts a more significant effect on global transcription in strain SN2 than does pyruvate. As expected, in comparisons where both growth conditions (habitats and carbon sources) differed, the correlation between transcriptional profiles was lower, compared to when only one growth condition varied (Fig. 2e,f).

**Figure 3:**
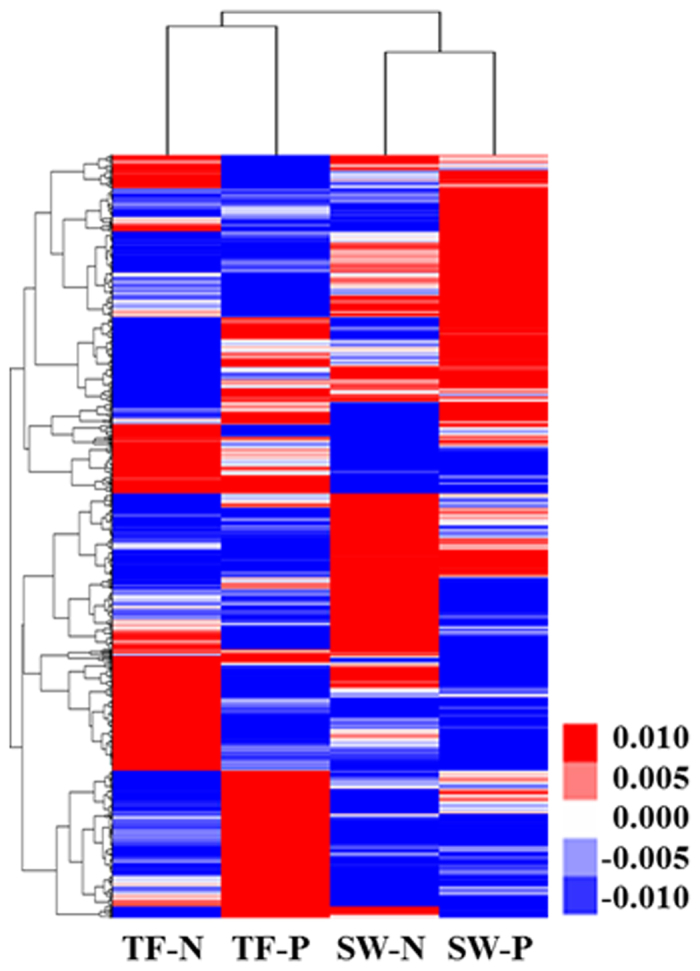
Hierarchical clustering showing the correlations between genome-wide transcriptional patterns of strain SN2 in TF-N, TF-P, SW-N, and SW-P conditions. The heat map was generated by Java TreeView (ver. 1.1.6r4) using mean-centered data of RPKM values. TF, tidal flat; SW, seawater; P, pyruvate; N, naphthalene.

Correlations between transcriptional profiles of strain SN2 from the four test conditions were displayed by hierarchical clustering using mean-centered data of RPKM values. Interestingly, the transcriptional profiles clustered by habitat (tidal flat or seawater), rather than by carbon sources (naphthalene or pyruvate), suggesting that the former may have greater effect than the latter on genome-wide transcription of strain SN2 (Fig. 3).

### Genome-wide functional analysis of gene expressions in strain SN2

**Figure 4:**
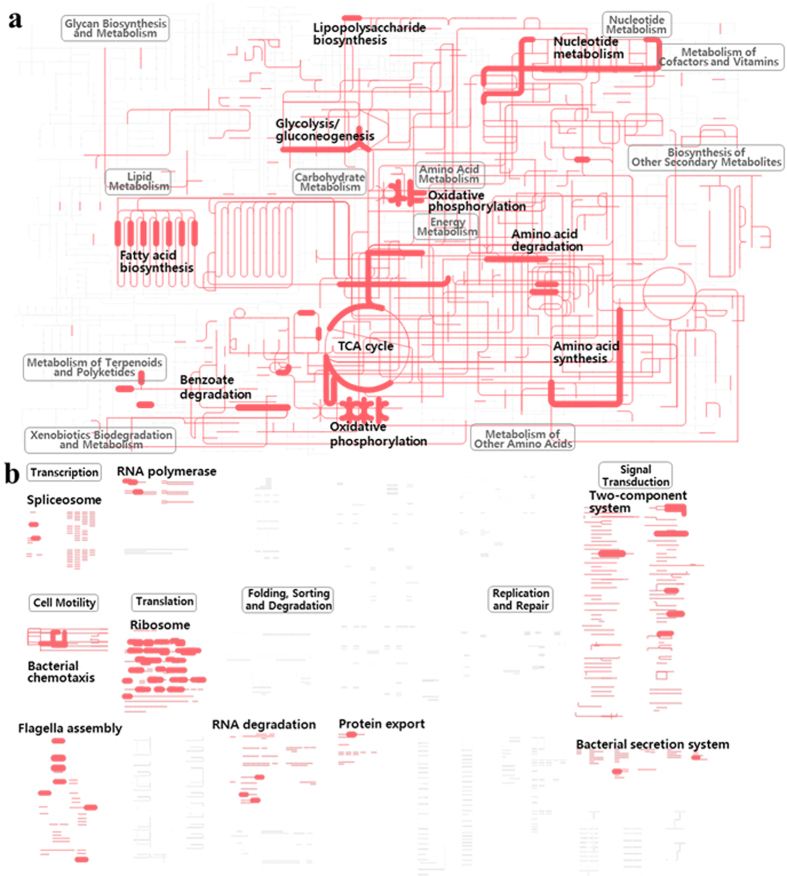
Metabolic (**a**) and regulatory (**b**) pathways showing elevated and constitutive gene expression in strain SN2. Pathways were generated using the iPath v2 module based on KEGG annotation of genes detected from sequencing. The thick nodes indicate metabolic enzymes or regulatory genes with RPKM values greater than 9 (based on a log_2_ scale) in all environmental test conditions, while the thin lines indicate the metabolic or regulatory pathways to which they related.

Genome-wide functional analysis of gene expression in the four environmental test conditions was carried out by metabolic mapping of clean cDNA reads onto KEGG pathways of strain SN2 using the iPath v2 module (Supplementary Fig. 3). The general mapping profiles of metabolic and regulatory pathways were similar in all test conditions, although transcriptional levels relating to some metabolic and regulatory pathways was found to differ to some degree (Fig. 4). Comparisons of metabolic mapping profiles derived from the test conditions showed that metabolic and regulatory genes associated with energy metabolism, translation, and cell motility, including those related to fatty acid biosynthesis, the tricarboxylic acid (TCA) cycle, oxidative phosphorylation, amino acid metabolism, proteins involved in the ribosome complex, bacterial chemotaxis (cheZ and cheY), flagella assembly, and two-component systems were highly expressed in all test conditions (Supplementary Table 1). The metabolic and regulatory pathways of strain SN2 for which high gene expressions was observed in all four test conditions correspond relatively well to those previously identified as showing elevated expression in *Alteromonas* species in response to environmental perturbations, such as a rapid increase in organic carbon sources^11^,^12^,^31^. In particular, sequences indicating active cell metabolism, including TCA cycle-related genes encoding citrate synthase, α-ketoglutarate dehydrogenase, succinyl-CoA synthase, isocitrate dehydrogenase, and malate dehydrogenase were highly expressed in all test conditions, as were protein synthesis-related genes encoding 30S and 50S ribosomal subunits, translation initiation factor IF-2, and elongation factors. These results suggest that the test conditions used in this study may have resulted in rapid utilization of the added organic substrates (naphthalene and pyruvate) by *Alteromonas*; thus, strain SN2 likely demonstrated *r*-strategist-like responses in the test conditions, similar to other *Alteromonas* species^32^,^33^. However, many of the highly expressed metabolic and regulatory genes found here for strain SN2 have previously exhibited elevated gene expression levels (high RPKM values) compared to other genes even under starvation conditions^11^,^31^. This may suggest that such genes are constitutively highly expressed, rather than being indicative of copiotrophy in *Alteromonas*.

### Genome-wide differential gene expressions in strain SN2 under four different environmental conditions

**Figure 5:**
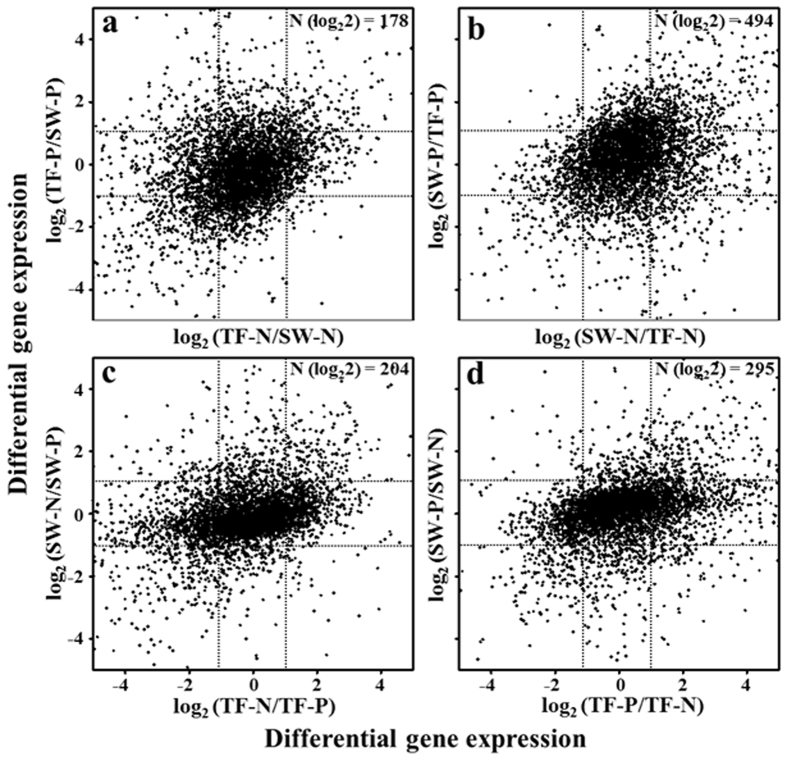
Differential gene expressions of strain SN2 in TF-N, TF-P, SW-N, and SW-P test conditions. Dots were calculated by comparing the RPKM values of genes in each condition, and dotted lines represent two-fold cutoffs signifying differential expression. Genes showing a more than two-fold differential expression on both axes were considered as highly and specifically expressed genes in response to the respective environmental variables, i.e. tidal flat (**a**), seawater (**b**), naphthalene (**c**), and pyruvate (**d**). The number of genes in this manner is given in the top right quadrant of each plot. TF, tidal flat; SW, seawater; P, pyruvate; N, naphthalene.

Although the overall gene expression profiles from TF-N, TF-P, SW-N, and SW-P conditions had many similarities, numerous genes were differentially and specifically up-regulated. Differential expressions of all CDSs in TF-N and TF-P environmental test conditions were displayed using two-dimensional coordinate systems (Fig. 5), and genes with differential expression more than two-fold (>log_2_ 2; on both axes of Fig. 5) were considered as being specifically up-regulated in response to the variables in question (tidal flat, seawater, naphthalene, and pyruvate). Fewer genes were differentially expressed in tidal flat (178) and naphthalene (204) conditions than in seawater (494) and pyruvate (295) conditions (Supplementary Table 2–5), suggesting that in tidal flat and naphthalene conditions, strain SN2 may employ more specific and limited metabolic and regulatory pathways.

**Figure 6:**
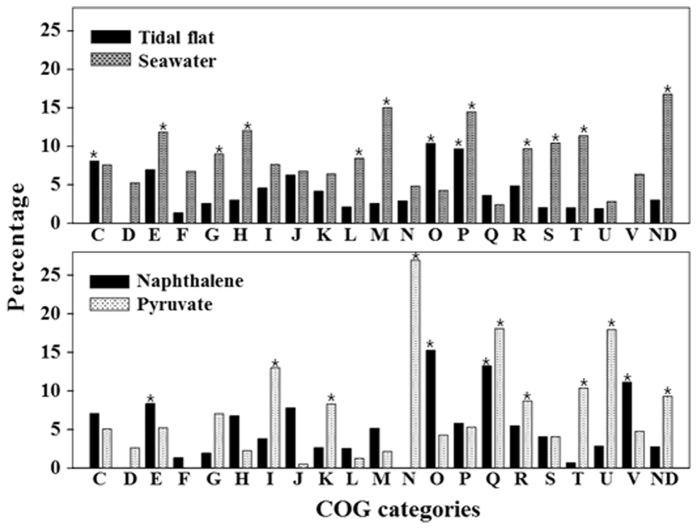
Clusters of orthologous groups (COG)-based functional classification of the specifically up-regulated genes identified in Fig. 7 in tidal flat, seawater, naphthalene, and pyruvate environments. The COG categories used are as follows: (C) energy production and conversion; (D) cell division and chromosome partitioning; (E) amino acid transport and metabolism; (F) nucleotide transport and metabolism; (G) carbohydrate transport and metabolism; (H) coenzyme transport and metabolism; (I) lipid transport and metabolism; (J) translation, including ribosomal structure, and biogenesis; (K) transcription; (L) replication, recombination, and repair; (M) cell wall, membrane, and envelope biogenesis; (N) cell motility; (O) post-translational modification, protein turnover, and chaperones; (P) inorganic ion transport and metabolism; (Q) secondary metabolite biosynthesis, transport, and catabolism; (R) general functional prediction only; (S) function unknown; (T) signal transduction mechanisms; (U) intracellular trafficking, secretion, and vesicular transport; (V) defense mechanisms; ND, not defined. Asterisks represent COG categories into which more than 8% of the total number of up-regulated genes were classified.

Those genes showing specific expression in response to the four environmental variables, tidal flat, seawater, naphthalene, and pyruvate, in Fig. 5 were classified into COG categories (Fig. 6). Regarding the tidal flat habitat, more percentages of genes of the O, P, and C categories showed specifically increased expression compared to those of other COG categories, while when exposed to seawater, more percentages of genes of many categories including M, P, E, and H were found to be up-regulated. Although the number of genes showing specifically elevated expression in tidal flat conditions (178) was much smaller than that observed in response to seawater (494), more genes of the O and C categories were specifically increased in the former compared to the latter. Thus, genes associated with post-translational modification, protein turnover, chaperones, and energy production and conversion may be specifically up-regulated in tidal flat habitats compared to seawater environments. In response to naphthalene, elevated expression of more percentages of genes in categories O, Q, V, and E were observed compared to other COG categories, while following exposure to pyruvate, more percentages of genes of the N, Q, U, I, T, and K categories were found to be up-regulated. A greater number of genes in categories O, J, V, H, E and C showed specifically increased expressions in response to naphthalene compared to pyruvate, while more genes of the N, U, T, K, I, and G categories were up-regulated in response to pyruvate compared to naphthalene. These results suggest that the expression of genes associated with post-translational modification, protein turnover, and chaperones, translation, and ribosomal structure and biogenesis may be increased as a consequence of being subjected to naphthalene rather than pyruvate, while genes associated with cell motility, intracellular trafficking, secretion, and vesicular transport, and signal transduction mechanisms may be up-regulated in response to pyruvate, when compared to naphthalene.

### Metabolic mapping of strain SN2 genes showing differential expressions under four environmental conditions

**Figure 7:**
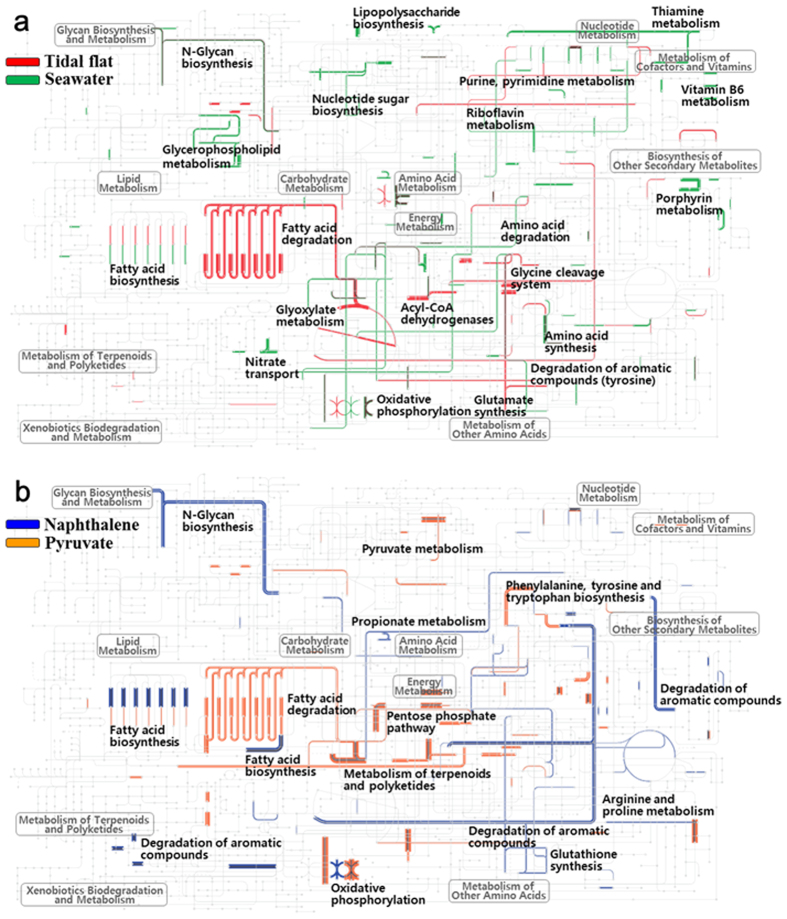
Metabolic pathways of strain SN2’s genes specifically up-regulated in tidal flat and seawater (**a**) and naphthalene and pyruvate (**b**) conditions. Pathways were generated using the iPath v2 module based on KEGG annotation of those genes identified in Fig. 7 as showing specific differential expression. Line widths are proportional to the expression levels of metabolic pathways.

To investigate the ecophysiological properties of strain SN2 in regard to the four different environmental variables, the metabolic pathways associated with genes showing increased expression in tidal flat, seawater, naphthalene, and pyruvate conditions were identified by metabolic mapping of the corresponding cDNA reads onto KEGG pathways using the iPath v2 module (Fig. 7).

This analysis showed that metabolic genes associated with glyoxylate metabolism, fatty acid degradation, oxidative phosphorylation, purine and pyrimidine metabolism, riboflavin metabolism, glycine cleavage system, aromatic compound metabolism, and glutamate synthesis (glutamate dehydrogenase) were highly expressed in response to the tidal flat habitat. In seawater, however, genes associated with *N*-glycan biosynthesis, glycerophospholipid metabolism, cell wall biogenesis, nucleotide sugar biosynthesis, thiamine metabolism, purine and pyrimidine metabolism, fatty acid biosynthesis, nitrate transport, oxidative phosphorylation, vitamin B_6_ metabolism, porphyrin metabolism, and amino acid synthesis were up-regulated (Fig. 7a). Besides genes that mapped onto strain SN2 KEGG pathways, those encoding stress response proteins (including phage shock protein, ClpB, heat shock proteins [hsp90 and GrpE], and chaperone proteins [GroEL, GroES, DnaJ, and DnaK]), twitching motility proteins (PilT and PilU), and phenylpropionate dioxygenase and related ring-hydroxylating dioxygenases showed elevated expressions in response to tidal flat (Supplementary Table 2). Meanwhile, genes encoding chromosomal replication initiator protein (DnaA), DNA polymerase III, glutamine synthetase, outer membrane protein (OmpW), TonB-dependent receptor, proteases, peptidases, lipase/acylhydrolase, nitrate and nitrite reductases, formate/nitrite transporters, cyanate hydratase, carbonic anhydrase, translation initiation factor IF-1, RNA polymerase sigma factors, catalase, superoxide dismutase, and polysaccharide biosynthesis/export proteins demonstrated increased expression in response to seawater (Supplementary Table 2).

Stress response and chaperone proteins play an essential role in the synthesis, transport, folding, and degradation of proteins to prevent unfavorable aggregation and to assist in proper refolding of damaged proteins. When microorganisms are exposed to environmental or chemical stresses including pollutants, they are known to increase production of several stress response and chaperone proteins for the protection of cells from the stresses^26^,^28^,^34^. Our differential expression analysis showed that many genes encoding stress response and chaperone proteins were highly expressed in strain SN2 under tidal flat conditions, suggesting that, compared to seawater, cells of this strain are exposed to a greater level of stress and demonstrate an increased metabolic potential for the degradation of organic chemicals, including aromatic compounds, in this environment. In addition, the elevated expression of these proteins in this habitat likely also contributes to the ecological fitness traits of strain SN2, enabling survival in seasonally cold marine tidal flats^6^. The genes *pilU* and *pilT*, distantly apart from the *pil* operon in the genome of strain SN2, and related to the elongation and retraction of pili important for ‘twitching’ or ‘gliding’ motility^35^, were expressed at increased levels in response to tidal flat conditions, implying that cells of this strain may more readily colonize by biofilm formation in tidal flats than in seawater. These results may partially support the previous finding that strain SN2 degrades naphthalene much more quickly in tidal flat slurry than in seawater^19^.

Increased expression of glutamine synthetase gene observed in seawater environments is consistent with previous findings that nitrogen two-component systems typically sense and respond to nitrogen limitation by activating glutamate metabolism^36^. This may explain why strain SN2 grows well and degrades naphthalene quickly despite a deficiency of nitrogen sources in seawater^19^. A gene encoding glutamate dehydrogenase, which has a high K_m_ value (i.e., a low affinity) for ammonia, was highly expressed in response to tidal flat conditions, perhaps owing to the higher concentration of ammonia in tidal flat sediment compared to seawater^37^. Unlike seawater, tidal flats include anaerobic conditions, meaning that nitrogen sources are present as ammonia in sediment, while in seawater they mainly exist in oxidized forms such as nitrate. This fact may further help to explain the increased expression of glutamate dehydrogenase gene in tidal flat samples. In addition, this may provide an explanation for the up-regulation in seawater of genes encoding enzymes involved in reactive oxygen species removal, such as catalase and superoxide dismutase, as well as those producing nitrite/nitrate-utilizing enzymes, such as formate/nitrite transporters, those involved in nitrate transport, and nitrate and nitrite reductases.

Metabolic mapping analysis showed that metabolic genes associated with *N*-glycan and fatty acid biosynthesis, oxidative phosphorylation, arginine, proline, and propionate metabolism, glutathione synthesis, and degradation of aromatic compounds were expressed at higher levels in response to naphthalene, while expression of those associated with fatty acid degradation, the pentose phosphate pathway, oxidative phosphorylation, and metabolism of pyruvate, terpenoids, polyketides, phenylalanine, tyrosine, and tryptophan increased in response to pyruvate (Fig. 7b). These results clearly support the previous findings that strain SN2 is capable of biodegrading aromatic hydrocarbons. Besides genes that mapped onto strain SN2 KEGG pathways, others encoding aromatic compound degradation-related proteins, cation/multidrug efflux pump, cytochrome *b*561, indole-3-glycerol phosphate synthase, Co/Zn/Cd efflux system component, thiol-disulfide isomerase and thioredoxins, and long-chain fatty acid transport protein showed higher expression in response to naphthalene. Meanwhile, genes encoding for Tfp pilus assembly protein, major pilin, TonB-dependent receptor, a stress response protein (CspD), chemotaxis response-related proteins (e.g., methyl-accepting chemotaxis protein), flagella-associated proteins, exopolyphosphatase-like protein, and restriction endonucleases were highly expressed after exposure to pyruvate. It has been well established that naphthalene and its metabolites induce chemotaxis and flagellar motility in microbes^38^,^39^, but some reports have indicated that pyruvate can also strongly trigger these responses^38^. Proteins related to chemotaxis and flagella were expressed highly in response to pyruvate in strain SN2, which may imply a stronger chemotactic response to pyruvate than naphthalene in this microbe.

### Expression of PAH degradation genes in strain SN2 under different environmental conditions

**Figure 8:**
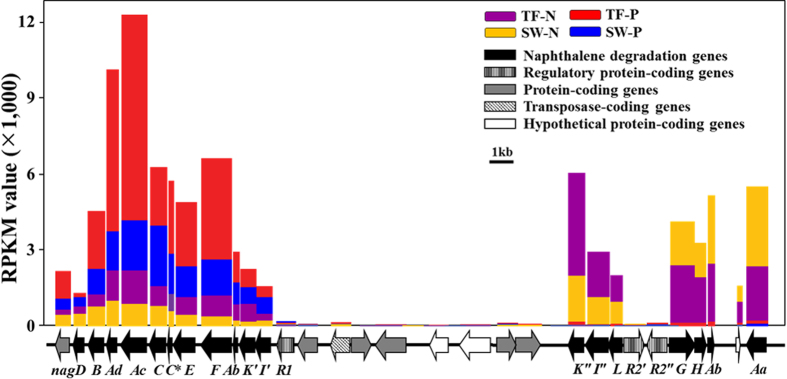
Comparison of transcriptional expressions of genes responsible for naphthalene degradation under four different environmental conditions. cDNA reads were mapped onto the coding sequences of putative naphthalene-degrading gene clusters; expression levels are indicated as RPKM values in a non-cumulative way. The black arrows indicate putative genes involved in naphthalene metabolism (nagC* is a partial gene of *nagC*). R1 and R2 are genes encoding GntR- and LysR-type regulatory proteins, respectively. Bars showing RPKM values in the four environmental test conditions are placed above the corresponding sequences.

Strain SN2 metabolizes naphthalene via the gentisate pathway and harbors naphthalene-catabolizing genes in two gene clusters present in a genomic island^6^. Moreover, these clusters contain uniquely genes encoding three regulatory proteins (one GntR-type and two LysR-type transcriptional regulators) that may be associated with the regulation of naphthalene-catabolizing genes in response to naphthalene or its metabolites (e.g., salicylate). However, the expressional property of these naphthalene-related gene clusters has not yet been established for strain SN2, although it is presumed that these catabolic genes are highly expressed in the presence of naphthalene. Therefore, their transcriptional characteristics were investigated by mapping cDNA reads derived from the four environmental test conditions onto the naphthalene-catabolizing gene sequences (Fig. 8). Genes encoding enzymes catabolizing from salicylate to central metabolites (*nagK“I“LGHAbAa*), including salicylate 5-hydroxylase (encoded by *nagGH*) and gentisate 1,2-dioxygenase (encoded by *nagI*”), probably controlled by the two LysR-type regulators (*R2’* and *R2”*), were highly up-regulated in response to naphthalene compared to pyruvate. However, genes encoding enzymes catabolizing naphthalene to salicylate (nagDBAdAcCC*EFAbK’I’), including naphthalene dioxygenase (encoded by *nagAcAd*), probably controlled by the GntR-type regulator (*R1*), were down-regulated under naphthalene conditions, compared to those including pyruvate. These results are not consistent with the transcriptional characteristics of the naphthalene-catabolizing genes identified previously well-studied naphthalene degraders, *Ralstonia* sp. U2 and *Polaromonas naphthalenivorans* CJ2, which also metabolize naphthalene via the gentisate pathway. In these strains, the genes encoding salicylate 5-hydroxylase and naphthalene dioxygenase are both controlled by a LysR-type regulator and are up-regulated in the presence of naphthalene^30^,^40^. As PAH degradation is not a common attribute in *Alteromonas* species, gene clusters conferring this ability were presumably only recently introduced into the genome of strain SN2 via a horizontal gene transfer, which may be also supported by the presence of the naphthalene degradation clusters in a genomic island. Therefore, it is possible that the organization of these clusters in strain SN2 may not be as finely regulated as that in *Ralstonia* sp. U2 and *P. naphthalenivorans* CJ2.

Although the expression of nagDBAdAcCC*EFAbK’I’ was lower (approximately 20%) under the naphthalene conditions than under the pyruvate conditions, the expression levels were relatively high compared to those of other genes in strain SN2. Strain SN2 rapidly degrades PAH compounds including naphthalene, phenanthrene, anthracene, and even pyrene in marine environments^19^. Therefore, these results suggest that the expression levels may be enough for the efficient PAH degradation by strain SN2. The gene *nagGH*, encoding salicylate 5-hydroxylase, is found from the upstream of *nagAcAd*, which encodes naphthalene dioxygenase, in the naphthalene-metabolizing gene clusters of *Ralstonia* sp. U2 and *P. naphthalenivorans* CJ2. Taken together, these observations also may suggest that naphthalene hydroxylation by naphthalene dioxygenase is a less critical step in naphthalene degradation than the conversion of salicylate to gentisate by salicylate 5-hydroxylase for efficient naphthalene degradation. On the other hand, the mapping analysis showed that the expression of nagDBAdAcCC*EFAbK’I’ was higher under the tidal flat conditions than under the seawater conditions, which strongly supports the previous results that strain SN2 degrades naphthalene much more efficiently in tidal flat than in seawater^19^.

In conclusion, a comprehensive analysis of the specific transcriptional cellular responses of strain SN2 under four environmental mimic conditions (namely, tidal flat-naphthalene, tidal flat-pyruvate, seawater-naphthalene, and seawater-pyruvate) revealed strain SN2 to be an opportunistic marine *r*-strategist. We also documented the expression of certain ecological fitness traits--with strain SN2 showing an appreciable capacity to degrade pollutants, including PAH compounds, in seasonally cold tidal flat habitats. Genome-wide transcriptional analysis, described here, can to add to the growing body of knowledge aiming to understand the ecophysiological properties and environmental behavior of bacteria in their natural habitats.

## Methods

### Collection of marine tidal flat sediment and seawater

Marine tidal flat sediment and seawater samples were collected in January 2014 from the Taean coast of South Korea (36°48'50.82″N,126°11'09.56″E), an area heavily contaminated by the *MV Hebei Spirit* oil spill of December 7, 2007, where *Alteromonas naphthalenivorans* SN2 (KACC 18427) was isolated^8^,^19^. The samples were immediately transported to the laboratory in an icebox and their PAH (specifically naphthalene, phenanthrene, anthracene, and pyrene) concentrations were measured by HPLC equipped with a fluorescence detector, as described previously^41^. The abundance of total bacteria and strain SN2 in tidal flat sediment and seawater samples was enumerated by quantitative real-time PCR (qPCR) based on the 16S rRNA gene using a CFX96^TM^ Real-Time PCR Detection System (Bio-Rad, Hercules, CA, USA) as described previously^19^,^42^. Universal (340F and 758R) and *Alteromonas*-specific (Alt845F and Alt1252R) primer sets were used to establish the quantity of *Bacteria* and *Alteromonas* species, respectively^19^.

### Preparation of four environmental mimic conditions and growth of strain SN2

Four environmental mimic test conditions, namely tidal flat-naphthalene (TF-N), tidal flat-pyruvate (TF-P), seawater-naphthalene (SW-N), and seawater-pyruvate (SW-P), were prepared for use in genome-wide transcription analysis. For the inoculation, cells of strain SN2 were cultivated in marine broth (BD, Detroit, MI, USA) at 25 °C with shaking at 180 rpm, and were harvested by centrifugation during the exponential growth phase (at an optical density at 600 nm [OD_600_] of approximately 0.9). The harvested cells were washed once, and resuspended using autoclaved seawater to be approximately 1.0 × 10^9 ^cells/ml, and subsequently used as an inoculum for the four test conditions.

For the growth of strain SN2 under the TF-N condition, twenty-one 160-ml serum bottles (eighteen of ^12^C-naphthalene and three of ^13^C-naphthalene), containing 3.0 mg of ^12^C- or ^13^C-naphthalene in 10 ml of tidal flat slurry were prepared as described previously^19^. Briefly, 60 μl of hexane stock solution containing 5% (w/v) of naphthalene was injected into sterile 160-ml serum bottles using a syringe, and the hexane was allowed to completely evaporate over the course of 5 min. Approximately 10 ml of tidal flat slurry (made by mixing 13.5 g of fresh tidal flat sediment with 1.5 ml of seawater) was added into the bottles and cells of strain SN2 were inoculated for a final concentration of approximately 1.0 × 10^6 ^cells/g-slurry. The serum bottles were then incubated at 25 °C with shaking of 180 rpm and, and were manually mixed twice a day. Periodically, naphthalene concentrations and the abundance of total bacteria and *Alteromonas* species in three sacrificial ^12^C-naphthalene bottles were analyzed as described previously^19^. For mRNA extraction, after 9 h of incubation, the slurry from three ^13^C-naphthalene serum bottles was combined and centrifuged at 7000 rpm for 3 min to allow the supernatant to be removed. The precipitated sediment was then resuspended in 120 ml of RNAlater (Ambion^®^, Austin, TX, USA) and stored at 4 °C until the mRNA extraction was carried out.

For the growth of strain SN2 under the TF-P condition, twenty-one 160-ml serum bottles containing 0.03% (w/v) pyruvate in approximately 10 ml of sterile tidal flat slurry (made by mixing 13.5 g of autoclaved tidal flat sediment with 1.5 ml of autoclaved seawater) were prepared, and cells of strain SN2 were inoculated for a final concentration of approximately 1.0 × 10^6 ^cells/ml. The serum bottles were incubated at 25 °C with shaking of 180 rpm, and were manually mixed twice a day. Periodically, pyruvate concentrations were analyzed by HPLC (model LC-20A; Shimadzu, Kyoto, Japan) equipped with an RI detector (RID-20A, Shimadzu) and a Repromer H column (250 × 8 mm; Dr. Maisch-GmbH, Ammerbuch-Entringen, Germany) with 1 mM sulfuric acid as an eluent. For mRNA extraction, after 12 hr of incubation, the slurry from three serum bottles was combined together and centrifuged at 7000 rpm for 3 min to allow the supernatant to be removed. The precipitated sediment was then resuspended in 120 ml of RNAlater and stored at 4 °C until mRNA extraction was carried out.

For the growth of strain SN2 under SW-N and SW-P conditions, 1-litter Erlenmeyer flasks containing 200 ml of fresh seawater with 0.03% (w/v) naphthalene or 0.1% (w/v) pyruvate were prepared in triplicate for each condition. Cells of strain SN2 were inoculated for a final concentration of approximately 1.0 × 10^7 ^cells/ml and were incubated at 25 °C with shaking of 180 rpm. The growth of strain SN2 was monitored by OD_600_ measurements and 10 ml was taken from each of the three flasks during the exponential growth phase (at 9 and 15 h following inoculation for SW-N and SW-P conditions, respectively) and combined before cells were harvested by centrifugation. These harvested cells were then resuspended in 1 ml of RNAlater and stored at 4 °C until mRNA extraction was carried out.

### RNA extraction and mRNA purification

The sediment slurries from TF-N and TF-P test conditions and the cells from SW-N and SW-P test conditions were centrifuged to enable removal of the RNAlater solution. Total RNA from the precipitated slurries and cells was extracted using an RNA PowerSoil Total RNA Isolation Kit (MO BIO, Carlsbad, CA, USA) and a TRIzol Max Bacterial RNA Isolation Kit (Life Technologies, Carlsbad, CA, USA), according to the manufacturers’ instructions. mRNA was then purified from total RNA by depleting ribosomal RNA using a Ribo-Zero Magnetic Kit (Epicentre, Madison, WI, USA), following the manufacturer’s instructions.

In addition, mRNA extracted from TF-N samples containing ^13^C-naphthalene underwent isopycnic centrifugation with a cesium trifluoroacetate (CsTFA) gradient to enrich strain SN2 mRNA, using a protocol slightly modified from that described previously^43^,^44^. Briefly, 4.8 ml of CsTFA solution (MP Biomedicals, Santa Ana, CA, USA), 200 μL of deionized formamide (Sigma-Aldrich, St. Louis, MO, USA), and 800 μL of nuclease-free water (Ambion) containing 800 ng mRNA, were mixed to give a solution with a CsTFA density of approximately 1.80 g/ml. The mixed solution was then pipetted into a 6 ml polyallomer ultracrimp tube (Sorvall, New Castle, DE, USA) and centrifuged at 40,000 rpm using a TV-865 titanium vertical ultracentrifuge rotor (Sorvall), for 65 h at 20 °C. Twenty-eight ‘heavy’ to ‘light’ fractions, approximately 200 μL in volume, were collected by top displacement with water using a syringe pump (model KDS-101; KD Scientific Inc., Holliston, MA, USA) at a flow rate of 2.0 μL/s. Two volumes of isopropanol and 5 μg of glycogen (Ambion) were added to the fractionation tubes before centrifugation at 14,000 × *g* for 30 min to precipitate mRNA. The mRNA precipitate was washed once with 70% ethanol and resuspended in 30 μL of nuclease-free water containing 45 units of an RNase inhibitor (RNasin Plus, Promega, Madison, WI, USA). Relative mRNA concentrations in the gradient fractions were measured with reverse transcriptase qPCR (RT-qPCR) using the gyrBBAUP2 and gyrBBNDN1 primer set targeting DNA gyrase subunit B (*gyrB*) transcripts, as described previously^45^. Those mRNA fractions with CsTFA densities of approximately 1.81 to 1.85 g/ml, considered as ^13^C-‘heavy’ fractions, were combined prior to Illumina sequencing.

### Illumina sequencing and bioinformatic data analysis

The purified mRNA derived from TF-N, TF-P, SW-N, and SW-P conditions was sequenced in 2 × 101-base paired-end mode using an Illumina HiSeq 2000 instrument (Illumina, San Diego, CA, USA) at Macrogen (Seoul, Korea), according to the procedure described previously^46^. Raw complementary DNA (cDNA) sequencing reads were processed using the FASTX-Toolkit program (available at http://hannonlab.cshl.edu/fastx_toolkit/), with the fastx commands “fastx_artifacts_filter -v -Q 33 -i INPUT_FASTQ -o OUTPUT_FASTQ; fastx_trimmer -Q 33 -f 2 -i INPUT_FASTQ -o OUTPUT_FASTQ; fastq_quality_trimmer -Q 33 -t 23 -l 50 -v -i INPUT_FASTQ -o OUTPUT_FASTQ”. The first base of each sequencing read was removed regardless of quality, due to the presence of an ambiguous base (“N”) at this position in many of the reads. Nucleotide sequences with low quality scores (<23, error rate = 0.005) were trimmed at the beginnings and ends of the reads, and subsequently, short sequencing reads of less than 50 nucleotides were eliminated. The resulting high-quality sequencing reads were aligned to the genome of strain SN2 available in GenBank using Burrows-Wheeler Aligner (BWA) software^47^, with the BWA command “bwa mem -t 35 DATABASE INPUT_FASTQ > OUTPUT_SAM”, specifying a 2% mismatch criterion. Only cDNA reads that mapped onto coding sequences (CDSs) were used in further analysis. RPKM values (read numbers per kb of each CDS, per million mapped sequences) for the quantification of the relative gene expressions were calculated by normalizing CDS lengths and total cDNA read numbers mapped onto all CDSs using custom Perl scripts. The transcriptional profiles of strain SN2 in the four test conditions were compared by hierarchical clustering using mean-centered data of RPKM values for the respective genes, for which distance and similarity were calculated by Euclidean distance and complete-linkage clustering using Gene Cluster version 3.0. Metabolic mapping of cDNA reads was quantitatively performed using the iPath v2 module (http://pathways.embl.de/iPath2.cgi#) and metabolic pathways in the genome of strain SN2 in the Kyoto Encyclopedia of Genes and Genomes (KEGG), as described previously^48^,^49^. Gene expression levels in KEGG metabolic pathways were indicated by line width and color density based on RPKM values. Fold changes in gene expression levels among the four test conditions were calculated by comparing the RPKM values of all CDSs in the genome of strain SN2. Predicted products of genes showing high fold changes in response to the four environmental variables (tidal flat, seawater, naphthalene, and pyruvate) were classified into clusters of orthologous groups (COGs) using BLAST searches against the COG database (National Center for Biotechnology Information [NCBI]; ftp://ftp.ncbi.nih.gov/pub/COG/COG), with a COG assignment E-value of less than 10^−6^. Metabolic pathways in response to the four environmental variables were investigated by metabolic mapping of the cDNA reads corresponding to genes showing specifically increased differential expressions in the genome of strain SN2 in KEGG using the iPath v2 module.

### Nucleotide sequence accession number

The transcriptomic sequencing data are publicly available in the NCBI Sequence Read Archive under accession no. SRP062445.

## Additional Information

**How to cite this article**: Jin, H. M. *et al.* Genome-wide transcriptional responses of *Alteromonas naphthalenivorans* SN2 to contaminated seawater and marine tidal flat sediment. *Sci. Rep.* **6**, 21796; doi: 10.1038/srep21796 (2016).

## Supplementary Material

Supplementary Information

## Figures and Tables

**Table 1:** Summary of the data concerning cDNA sequencing reads derived from tidal flat-naphthalene (TF-N), tidal flat-pyruvate (TF-P), seawater-naphthalene (SW-N), and seawater-pyruvate (SW-P) test conditions.

	Test conditions			
	TF-N	TF-P	SW-N	SW-P
No. of raw cDNA reads (SRA No.)	10,919,820 (SRR2166618)	13,219,220 (SRR2170985)	13,188,062 (SRR2166612)	13,536,842 (SRR2166619)
No. of clean cDNA reads	10,643,078	13,030,928	13,015,794	13,371,743
No. (%*) of clean cDNA reads mapped onto the genome of strain SN2	5,887,843 (55.3)	4,643,259 (35.6)	12,970,339 (99.7)	13,182,147 (98.6)
No. (%*) of clean cDNA reads mapped onto strain SN2 CDSs	2,302,258 (21.6)	4,576,732 (35.1)	12,861,500 (98.8)	13,094,123 (97.9)

*The numbers in parenthesis indicate percentages for clean cDNA reads. cDNA, complementary DNA; CDS, coding sequence.
