# Supplementary material for: Genome-wide transcriptional responses of Alteromonas naphthalenivorans SN2 to contaminated seawater and marine tidal flat sediment
Source: Sci Rep. 2016 Feb 18;6:21796. doi: 10.1038/srep21796 (PMC4757865; doi:10.1038/srep21796)
Supplement: Supplementary Information [file srep21796-s1.pdf]

Supplementary information

Genome-wide transcriptional responses of *Alteromonas naphthalenivorans* SN2 to contaminated seawater and marine tidal flat sediment

Hyun Mi Jin[1,2], Hye Im Jeong[1], Kyung Hyun Kim[1], Yoonsoo Hahn[1], Eugene L. Madsen[3] & Che Ok Jeon[1,*]

[1]*Department of Life Science, Chung-Ang University, Seoul 06974, Republic of Korea*

[2]*Freshwater Bioresources Utilization Division, Nakdonggang National Institute of Biological Resources, Gyeongsangbuk-do 37242, Republic of Korea*

[3]*Department of Microbiology, Cornell University, Ithaca, NY 14853-8101, USA*

Supplementary figure legends

Supplementary Figure 1. Relative abundances of DNA gyrase subunit B (*gyrB*) transcripts recovered from cesium trifluoroacetate (CsTFA) gradient fractions. Expression of *gyrB* was determined by quantitative real-time PCR using mRNA from each gradient fraction, with transcript abundance being calculated relative to that of fraction 1.

Supplementary Figure 2. Frequency distribution of the expression levels of all coding sequences in the genome of strain SN2. Gene transcriptional levels ($\log_2$ RPKM values) are plotted on the X-axis, while the Y-axis represents the number of gens for which these levels were observed. Log_2-transformed RPKM values were placed into bins separated by increments of 1. TF, tidal flat; SW, seawater; P, pyruvate; N, naphthalene. RPKM, read number per kb of each coding sequence, per million mapped sequences.

Supplementary Figure 3. Metabolic and regulatory pathway mapping of clean cDNA reads corresponding to coding sequences in the genome of strain SN2 derived from the following conditions: (a) tidal flat with naphthalene, (b) tidal flat with pyruvate, (c) seawater with naphthalene, and (d) seawater with pyruvate. Metabolic and regulatory pathways were generated using the iPath v2 module based on Kyoto Encyclopedia of Genes and Genomes (KEGG) annotation of the genes detected from sequencing. Gene expression levels based on RPKM values are indicated on the KEGG pathways by line width and color density. RPKM, read number per kb of each coding sequence, per million mapped sequences.

Supplementary Figure 1

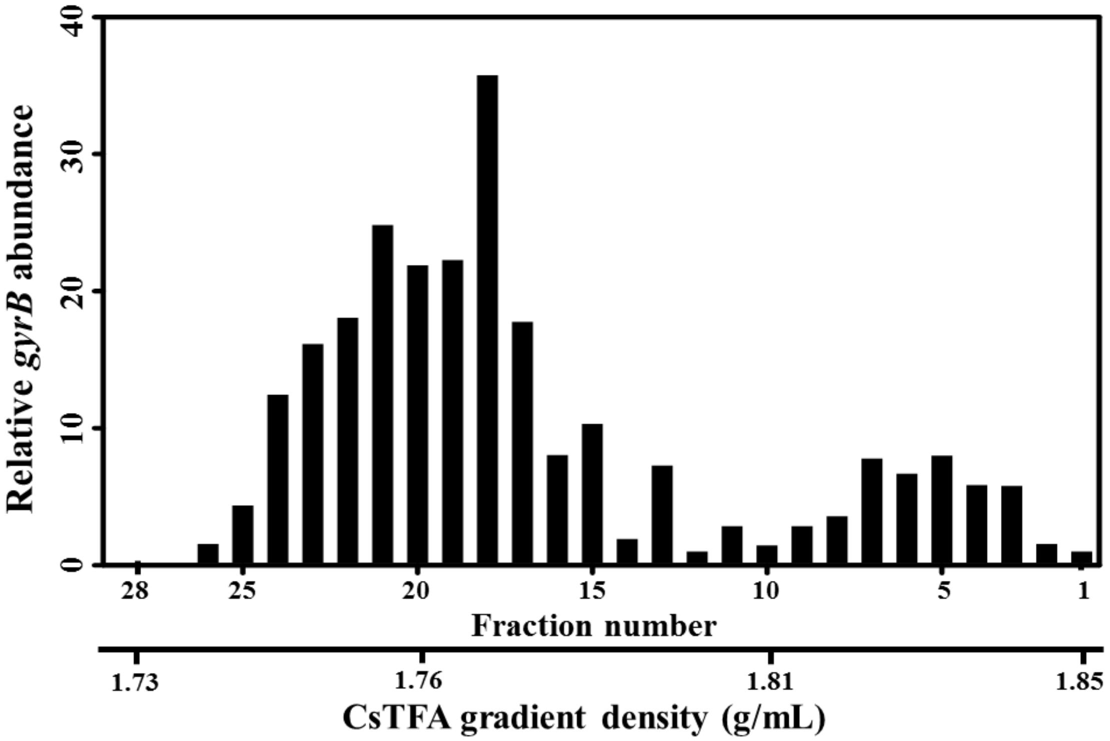

Supplementary Figure 2

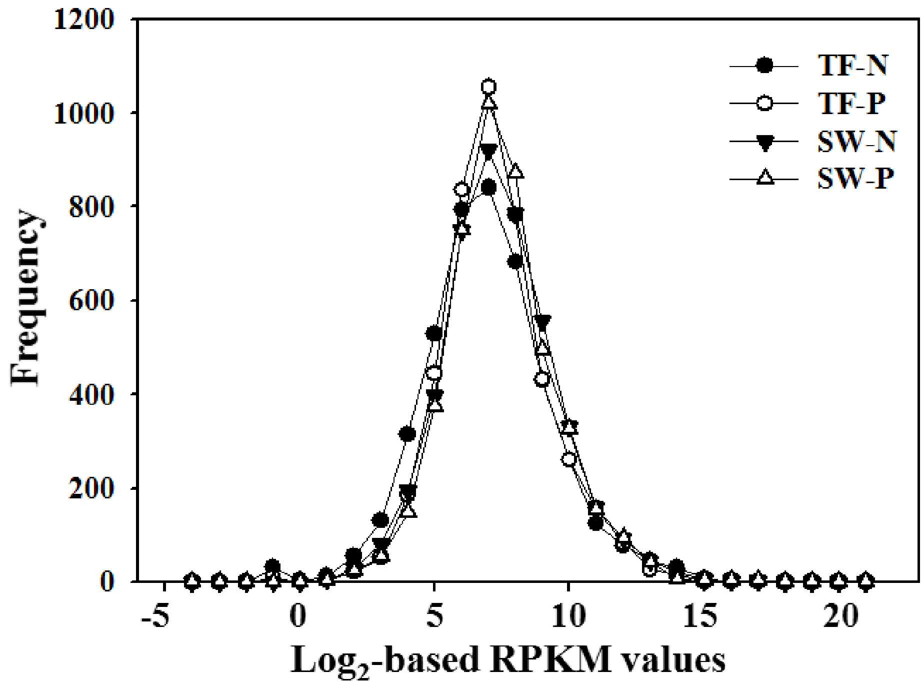

Supplementary Figure 3

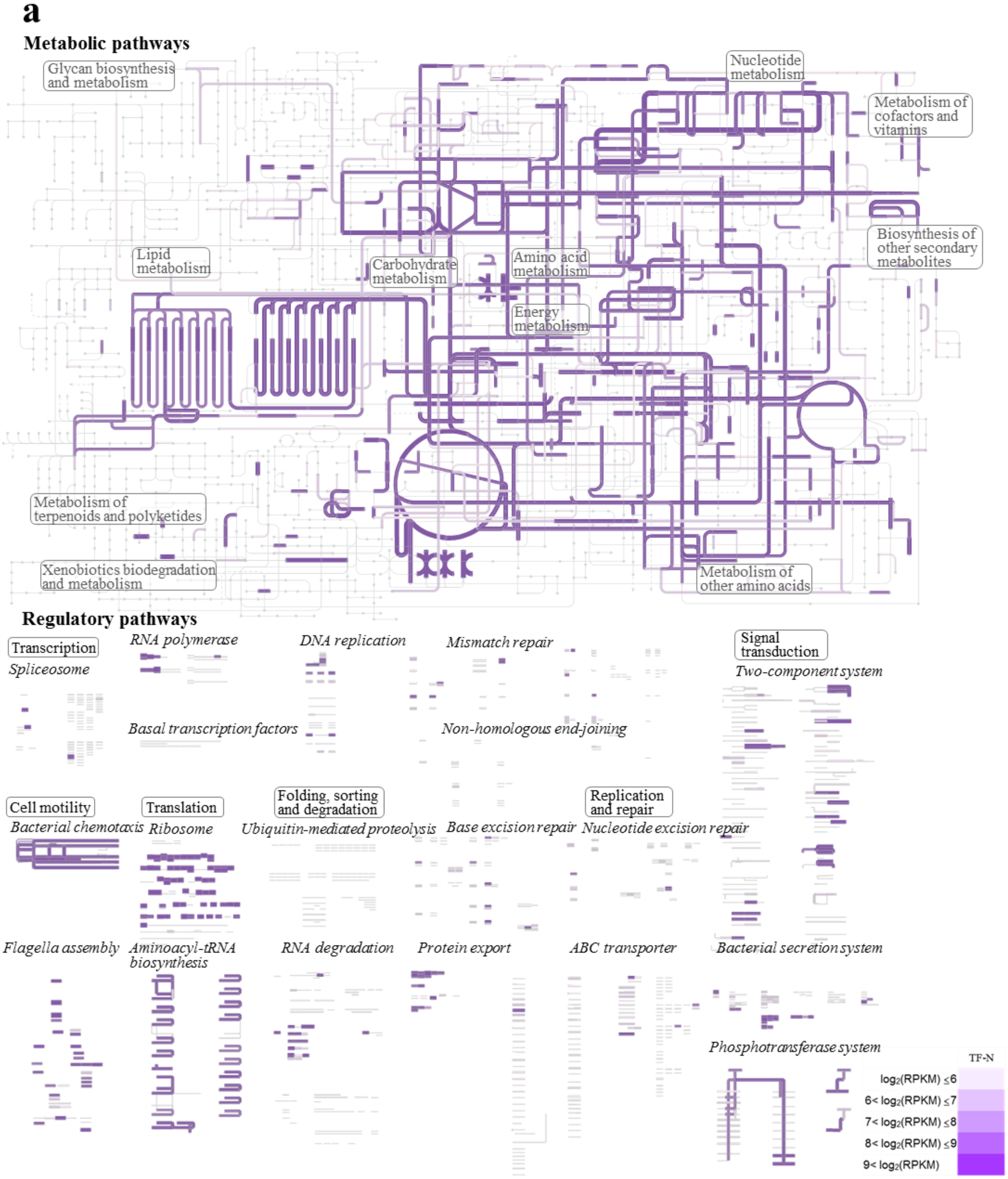

b

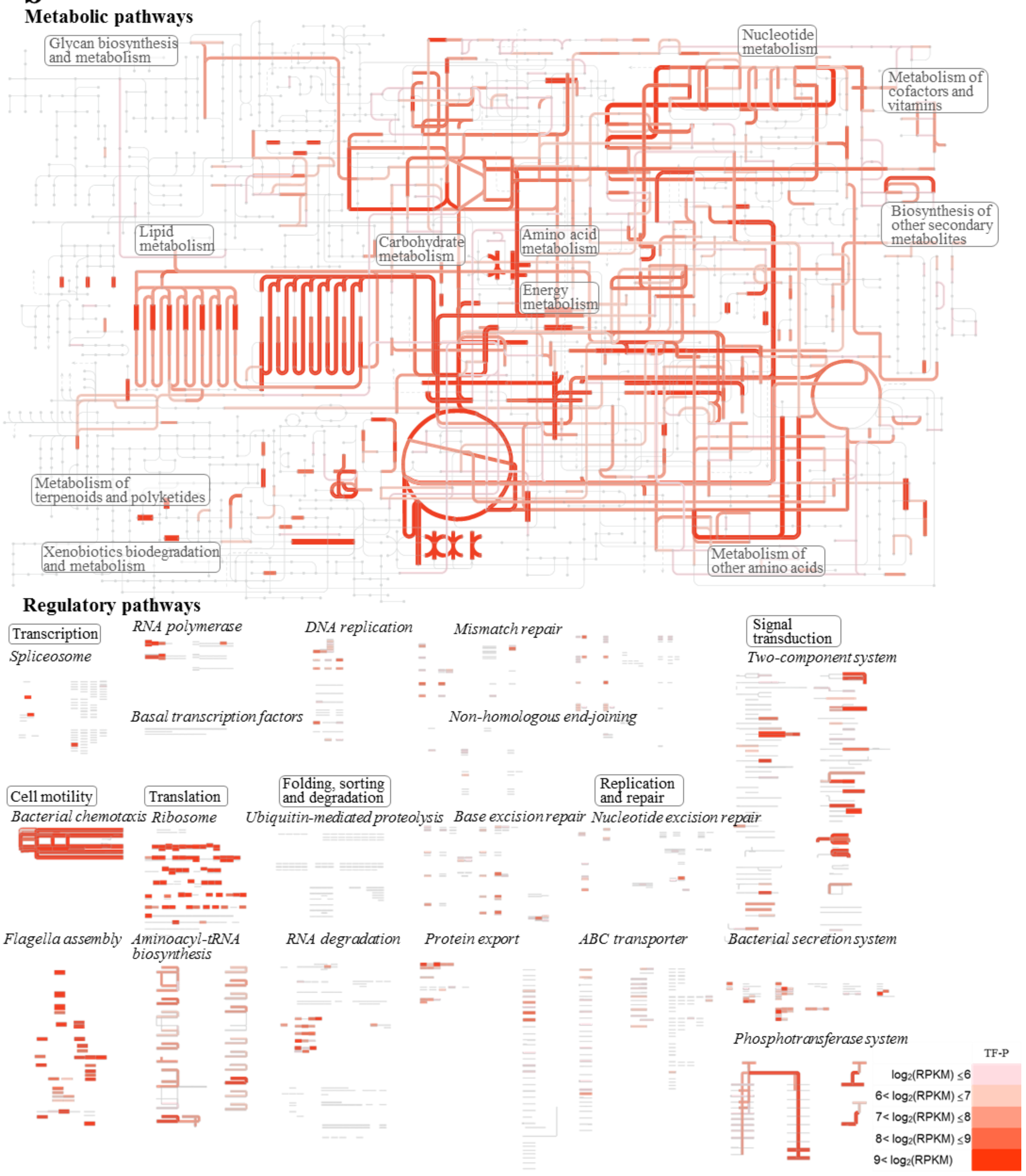
Metabolic pathways
Glycan biosynthesis and metabolism
Nucleotide metabolism
Metabolism of cofactors and vitamins
Biosynthesis of other secondary metabolites
Lipid metabolism
Carbohydrate metabolism
Amino acid metabolism
Energy metabolism
Metabolism of terpenoids and polyketides
Xenobiotics biodegradation and metabolism
Metabolism of other amino acids
Regulatory pathways
Transcription
Spliceosome
RNA polymerase
Basal transcription factors
DNA replication
Mismatch repair
Non-homologous end-joining
Signal transduction
Two-component system
Cell motility
Bacterial chemotaxis
Translation
Ribosome
Folding, sorting and degradation
Ubiquitin-mediated proteolysis
Base excision repair
Replication and repair
Nucleotide excision repair
Flagella assembly
Aminoacyl-tRNA biosynthesis
RNA degradation
Protein export
ABC transporter
Bacterial secretion system
Phosphotransferase system
TF-P
log2(RPKM) ≤6
6< log2(RPKM) ≤7
7< log2(RPKM) ≤8
8< log2(RPKM) ≤9
9< log2(RPKM)

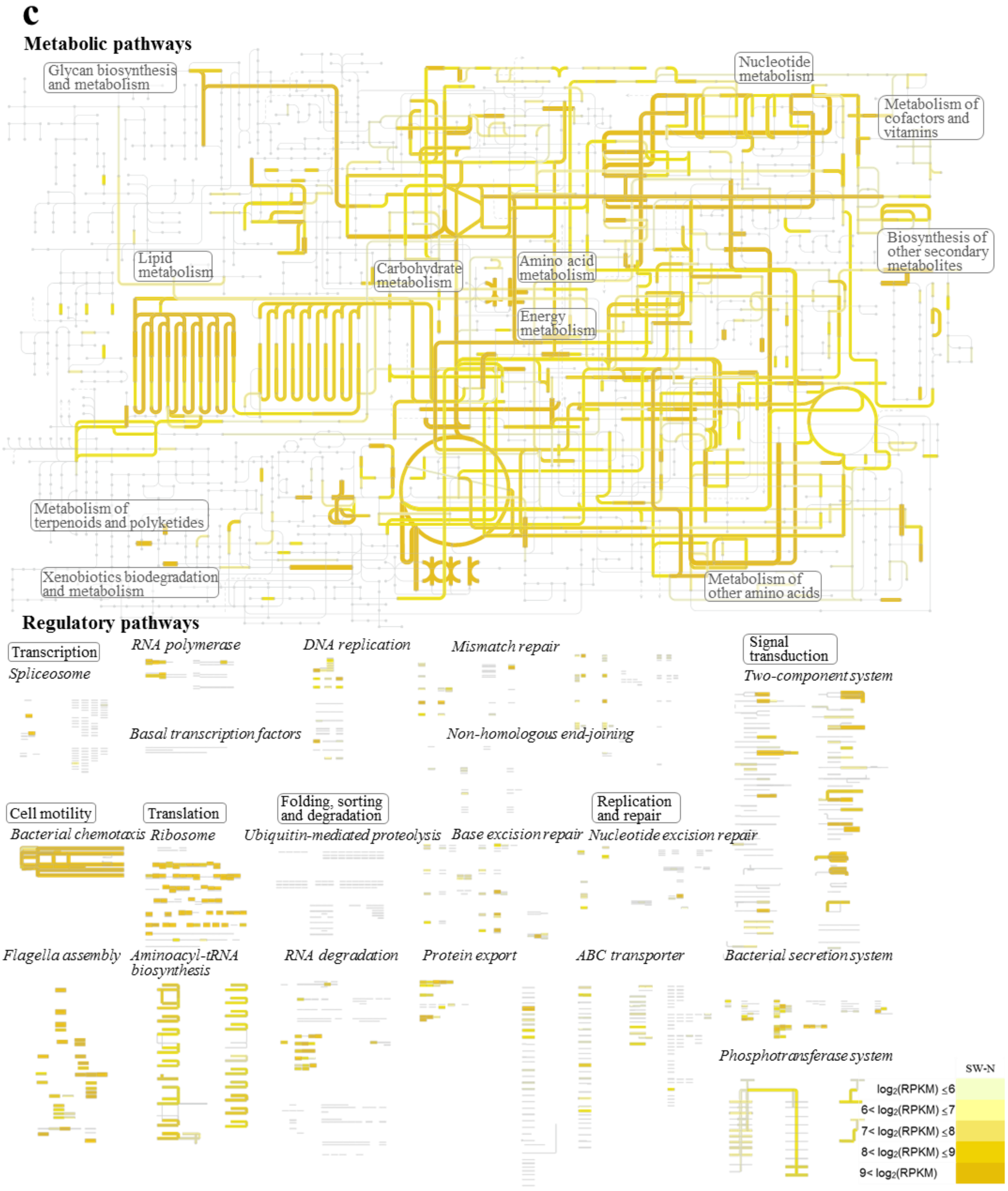
c
Metabolic pathways
Glycan biosynthesis and metabolism
Nucleotide metabolism
Metabolism of cofactors and vitamins
Lipid metabolism
Carbohydrate metabolism
Amino acid metabolism
Energy metabolism
Biosynthesis of other secondary metabolites
Metabolism of terpenoids and polyketides
Xenobiotics biodegradation and metabolism
Metabolism of other amino acids
Regulatory pathways
Transcription
Spliceosome
RNA polymerase
Basal transcription factors
DNA replication
Mismatch repair
Non-homologous end-joining
Signal transduction
Two-component system
Cell motility
Bacterial chemotaxis
Translation
Ribosome
Folding, sorting and degradation
Ubiquitin-mediated proteolysis
Base excision repair
Replication and repair
Nucleotide excision repair
Flagella assembly
Aminoacyl-tRNA biosynthesis
RNA degradation
Protein export
ABC transporter
Bacterial secretion system
Phosphotransferase system
SW-N
log2(RPKM) ≤6
6< log2(RPKM) ≤7
7< log2(RPKM) ≤8
8< log2(RPKM) ≤9
9< log2(RPKM)

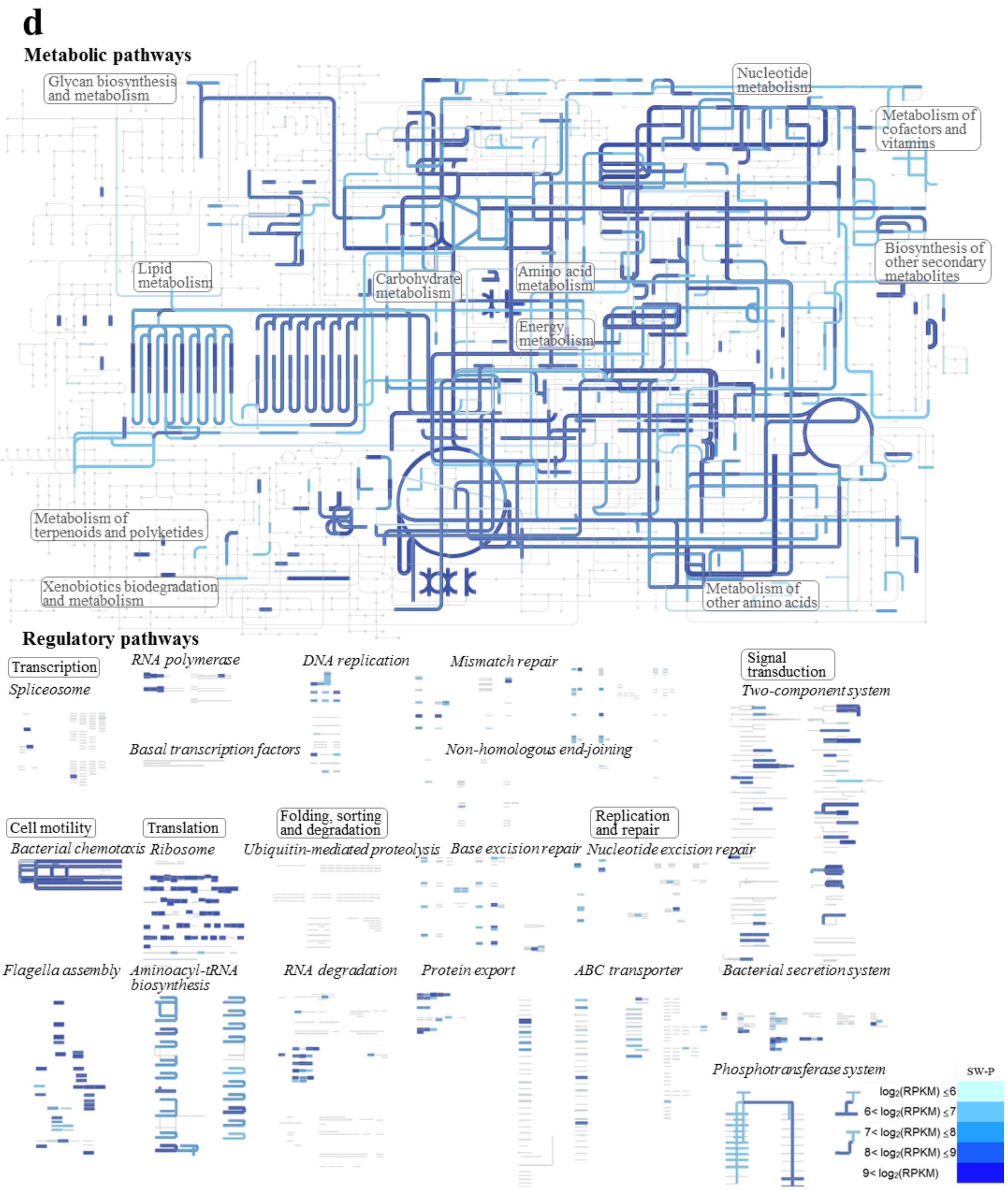
d
Metabolic pathways
Glycan biosynthesis and metabolism
Nucleotide metabolism
Metabolism of cofactors and vitamins
Lipid metabolism
Carbohydrate metabolism
Amino acid metabolism
Biosynthesis of other secondary metabolites
Energy metabolism
Metabolism of terpenoids and polyketides
Xenobiotics biodegradation and metabolism
Metabolism of other amino acids
Regulatory pathways
Transcription
Spliceosome
RNA polymerase
Basal transcription factors
DNA replication
Mismatch repair
Non-homologous end-joining
Signal transduction
Two-component system
Cell motility
Bacterial chemotaxis
Translation
Ribosome
Folding, sorting and degradation
Ubiquitin-mediated proteolysis
Base excision repair
Replication and repair
Nucleotide excision repair
Flagella assembly
Aminoacyl-tRNA biosynthesis
RNA degradation
Protein export
ABC transporter
Bacterial secretion system
Phosphotransferase system
SW-P
log2(RPKM) ≤6
6< log2(RPKM) ≤7
7< log2(RPKM) ≤8
8< log2(RPKM) ≤9
9< log2(RPKM)

Supplementary Table 1. Highly expressed strain SN2 genes with RPKM values (log_2-based) greater than 9 in all four environmental test conditions. TF, tidal flat; SW, seawater; P, pyruvate; N, naphthalene; RPKM, read number per kb of each coding sequence, per million mapped sequences.

Gene	Log_2-based RPKM value				Putative function
	TF-N	TF-P	SW-N	SW-P	
ambt_00205	13.41	12.61	11.04	10.80	Elongation factor Tu
ambt_00415	11.72	10.21	10.56	9.58	Malate dehydrogenase
ambt_00445	10.51	9.82	9.51	9.47	Na (+)-translocating NADH-quinone reductase subunit A
ambt_00450	10.33	9.69	9.25	9.24	Na (+)-translocating NADH-quinone reductase subunit B
ambt_00455	10.30	9.28	9.55	9.60	Na (+)-translocating NADH-quinone reductase subunit C
ambt_00460	10.13	9.76	9.20	9.36	Na (+)-translocating NADH-quinone reductase subunit D
ambt_00465	10.05	9.58	9.03	9.34	Na (+)-translocating NADH-quinone reductase subunit E
ambt_00470	10.54	9.86	9.40	9.63	Na (+)-translocating NADH-quinone reductase subunit F
ambt_00600	9.92	9.76	10.96	11.84	Biopolymer transport protein ExbD/TolR
ambt_01485	9.24	10.66	12.80	11.62	Glutamine synthetase
ambt_01490	9.39	9.15	9.61	9.58	BipA protein
ambt_02160	9.21	10.26	10.02	9.07	Cell division protein MraZ
ambt_02230	10.04	10.41	9.69	9.65	Cell division protein FtsZ
ambt_02235	9.72	9.60	10.57	9.80	UDP-3-O-[3-hydroxymyristoyl] N-acetylglucosamine deacetylase
ambt_02285	10.57	14.92	10.57	14.97	Type IV prepilin TapA
ambt_03675	10.56	9.95	11.71	10.49	hypothetical protein
ambt_04065	9.42	9.56	10.33	10.22	ATP-dependent protease ATP-binding subunit ClpX
ambt_04075	11.69	11.11	10.51	11.59	Histone-like DNA-binding protein
ambt_04125	11.76	9.88	12.08	11.50	30S ribosomal subunit protein S20
ambt_04260	9.03	10.72	12.22	11.90	Sigma E factor negative regulatory protein
ambt_06595	10.88	13.81	10.60	15.71	Hypothetical protein
ambt_06620	9.77	12.86	10.28	15.12	Hypothetical protein
ambt_06760	11.27	11.17	14.03	10.97	Peroxiredoxin
ambt_06990	9.37	9.12	10.19	9.47	HicB family protein
ambt_07550	10.50	10.51	9.93	10.36	Citrate synthase
ambt_07555	10.67	9.98	10.24	10.80	Succinate dehydrogenase, cytochrome b subunit
ambt_07560	10.83	10.19	9.83	10.39	Putative succinate dehydrogenase, hydrophobic subunit, cytochrome b556 with sdhC
ambt_07565	10.44	10.08	9.18	9.73	Succinate dehydrogenase flavoprotein subunit
ambt_07570	10.38	9.98	9.24	9.69	Succinate dehydrogenase catalytic subunit
ambt_07575	10.46	9.69	9.53	9.44	2-Oxoglutarate dehydrogenase E1 component
ambt_07580	11.26	10.37	9.64	9.68	Dihydrolipoyltranssuccinate transferase, component of the 2-oxoglutarate dehydrogenase complex
ambt_07585	12.26	10.68	9.31	9.91	Succinyl-CoA synthetase subunit beta
ambt_07590	11.89	10.30	9.07	9.72	Succinyl-CoA synthetase subunit alpha
ambt_08020	9.56	9.21	9.80	9.98	ProP expression regulator
ambt_08100	11.09	13.62	12.50	12.75	Ribosome modulation factor
ambt_08230	10.04	9.14	9.67	9.41	cbb3-type cytochrome c oxidase subunit I
ambt_08235	10.02	9.22	9.46	9.19	cbb3-type cytochrome c oxidase subunit II
ambt_08240	10.78	9.26	9.69	9.50	Cytochrome c oxidase subunit CcoQ
ambt_08245	9.97	9.24	9.33	9.08	Cytochrome c oxidase, cbb3-type subunit III
ambt_08420	10.71	9.19	10.35	9.63	hypothetical protein
ambt_08505	11.76	11.17	11.50	11.70	integration host factor subunit beta
ambt_08510	12.12	11.14	11.15	11.34	30S ribosomal protein S1
ambt_09400	10.21	9.22	9.70	10.40	polynucleotide phosphorylase/polyadenylase
ambt_09490	10.36	9.58	10.14	9.98	ATP-dependent metalloprotease FtsH
ambt_09655	9.64	10.00	9.27	9.08	ATP-dependent Clp protease ATP-binding protein ClpA
ambt_09660	9.84	9.30	9.63	9.29	ATP-dependent Clp protease adaptor protein ClpS
ambt_09665	9.58	12.50	12.78	14.28	Stress response protein CspD
ambt_09980	11.35	9.79	10.91	11.65	Acyl carrier protein
ambt_10005	12.22	10.67	12.34	11.83	50S Ribosomal protein L32
ambt_10010	13.31	10.85	13.06	12.61	Hypothetical protein
ambt_10685	12.39	10.95	12.51	12.45	50S Ribosomal protein L20
ambt_10690	13.63	12.52	13.51	13.48	50S Ribosomal protein L35

ambt_10695	12.25	11.30	12.60	12.63	Translation initiation factor IF-3
ambt_11110	11.44	10.88	10.79	10.92	Elongation factor G
ambt_12085	9.52	9.30	9.46	9.71	Peptidoglycan-associated lipoprotein
ambt_12235	13.25	9.71	10.85	10.25	tRNA (guanine-N(1)-)-methyltransferase
ambt_12240	13.57	10.24	11.32	10.78	16S rRNA-processing protein RimM
ambt_12245	13.78	10.42	11.50	11.07	30S Ribosomal protein S16
ambt_12345	11.97	12.55	10.59	12.25	Hypothetical protein
ambt_12575	10.33	9.30	12.74	12.46	Hypothetical protein
ambt_12635	12.68	11.09	11.38	11.97	Iron superoxide dismutase
ambt_12915	11.13	9.13	10.78	10.83	TonB family protein
ambt_12920	11.11	9.29	11.09	10.94	Biopolymer transport protein
ambt_12925	11.15	9.57	11.26	11.12	TonB system biopolymer transport protein
ambt_12930	11.18	10.58	11.36	11.28	MotA/TolQ/ExbB proton channel
ambt_12935	11.48	10.42	11.71	11.62	Hypothetical protein
ambt_12965	11.20	13.16	9.08	11.47	TonB-dependent receptor
ambt_13080	11.01	11.74	12.24	11.73	Carbon storage regulator
ambt_13195	9.81	10.26	10.07	10.77	Chemotaxis protein cheZ
ambt_13200	9.52	10.01	9.59	10.38	Chemotaxis protein CheY
ambt_13425	9.99	12.42	9.82	10.87	Flagellar hook-associated 2-like protein
ambt_13430	9.70	12.69	9.83	10.86	Flagellar protein FlaG protein
ambt_13435	10.53	14.94	9.90	11.79	Flagellin-like protein
ambt_13440	10.44	12.48	10.97	11.47	Hypothetical protein
ambt_13445	10.88	13.24	10.59	11.56	Flagellin-like protein
ambt_13450	13.46	15.67	12.05	13.96	Flagellin-like protein
ambt_13490	9.07	9.92	10.47	10.59	Flagellar basal-body rod protein FlgF
ambt_13495	9.03	11.46	11.02	11.21	Flagellar hook protein FlgE
ambt_13505	9.22	11.48	11.08	11.40	Flagellar basal-body rod protein FlgC
ambt_13530	10.04	11.85	9.94	11.07	Negative regulator of flagellin synthesis FlgM
ambt_13535	9.94	11.27	9.98	11.02	Putative flagellar protein FlgN
ambt_13660	10.08	9.17	10.62	10.83	OmpH family outer membrane protein
ambt_13705	12.46	10.41	11.29	11.07	30S Ribosomal protein S2
ambt_13755	9.38	11.36	15.28	16.70	Hypothetical protein
ambt_14385	10.86	9.29	10.03	11.10	Fructose-1,6-bisphosphate aldolase
ambt_14450	15.89	15.23	16.02	15.70	Hypothetical protein
ambt_14610	11.21	10.99	9.27	9.48	Ribosomal subunit interface protein
ambt_14680	10.88	10.33	9.08	9.40	Ubiquinol-cytochrome C reductase iron-sulfur subunit
ambt_14710	11.48	9.67	10.11	9.06	30S Ribosomal protein S9
ambt_14715	12.14	9.69	11.09	9.91	50S Ribosomal protein L13
ambt_15015	9.64	9.04	9.85	9.26	RNA polymerase sigma factor
ambt_15260	11.38	12.35	10.83	12.62	Hypothetical protein
ambt_15535	11.65	10.10	11.21	10.78	50S Ribosomal protein L27
ambt_15540	12.20	10.60	12.11	11.46	50S ribosomal protein L21
ambt_15830	9.48	9.60	12.09	10.27	Putative DNA-binding stress protein
ambt_15870	11.43	9.92	11.27	10.25	Thioredoxin
ambt_16500	12.83	9.64	9.83	9.07	50S Ribosomal protein L29
ambt_16505	13.02	11.56	10.56	9.58	50S Ribosomal protein L16
ambt_16515	12.90	10.69	10.90	9.27	50S Ribosomal protein L22
ambt_16520	12.61	10.55	10.80	9.27	30S Ribosomal protein S19
ambt_16530	12.37	10.23	10.83	9.03	50S Ribosomal protein L23
ambt_16540	12.26	10.56	11.24	9.68	50S Ribosomal protein L3
ambt_16545	12.75	10.40	12.23	10.70	30S Ribosomal protein S10
ambt_17825	10.82	10.34	11.11	11.73	Glutathione S-transferase
ambt_17885	10.29	12.15	9.58	11.14	2,3-Dihydroxy-2,3-dihydrophenylpropionate dehydrogenase
ambt_17890	11.12	13.31	9.96	11.87	Aromatic-ring-hydroxylating dioxygenase subunit beta
ambt_17895	11.12	13.59	9.77	12.02	Ring hydroxylating dioxygenase subunit alpha
ambt_17900	10.62	12.62	9.64	11.95	Protocatechuate 4,5-dioxygenase subunit beta
ambt_17905	10.32	12.49	9.27	11.48	Hypothetical protein
ambt_18080	10.04	9.18	9.89	11.31	Hypothetical protein
ambt_18610	9.51	9.85	10.83	10.94	Hypothetical protein
ambt_18750	11.26	10.85	12.06	12.30	Hypothetical protein
ambt_19040	11.57	9.33	9.93	9.61	50S Ribosomal protein L17
ambt_19045	12.56	10.71	11.13	10.79	DNA-Directed RNA polymerase subunit alpha

ambt_19050	12.85	11.09	11.71	11.30	30S Ribosomal protein S4
ambt_19055	12.41	11.76	11.38	10.99	30S Ribosomal protein S11
ambt_19060	13.02	11.11	12.31	11.85	30S Ribosomal protein S13
ambt_19065	13.21	11.96	12.43	12.11	50S Ribosomal protein L36
ambt_19070	12.41	10.73	10.87	10.58	Preprotein translocase subunit SecY
ambt_19075	12.56	10.35	10.74	10.10	50S Ribosomal protein L15
ambt_19080	12.92	11.19	11.30	10.71	50S Ribosomal protein L30
ambt_19085	12.26	11.01	10.41	9.72	30S Ribosomal protein S5
ambt_19090	11.95	9.84	10.09	9.43	50S Ribosomal protein L18
ambt_19095	12.35	10.44	10.75	9.91	50S Ribosomal protein L6
ambt_19100	12.04	9.83	10.12	9.15	30S Ribosomal protein S8
ambt_19105	12.51	10.66	10.79	9.91	30S Ribosomal protein S14
ambt_19110	12.62	11.21	11.13	10.31	50S Ribosomal protein L5
ambt_19115	12.58	11.10	11.14	10.45	50S Ribosomal protein L24
ambt_19120	13.21	10.76	12.32	11.64	50S Ribosomal protein L14
ambt_19240	12.86	12.07	13.77	14.54	Putative cold shock-like protein cspG
ambt_19365	11.16	9.53	11.54	10.55	50S Ribosomal subunit protein L31
ambt_19420	11.67	9.66	11.75	10.86	RNA Polymerase sigma factor
ambt_19610	13.09	12.61	11.09	11.07	Elongation factor Tu
ambt_19615	11.47	9.49	9.91	9.52	Translation elongation factor G
ambt_19620	13.37	10.80	11.21	10.74	30S Ribosomal protein S7
ambt_19625	13.50	11.53	11.83	11.38	30S Ribosomal protein S12
ambt_19635	10.46	10.31	9.92	9.20	DNA-Directed RNA polymerase subunit beta
ambt_19640	13.65	10.73	10.34	9.64	50S Ribosomal protein L7/L12
ambt_19645	14.04	11.16	11.29	10.38	50S Ribosomal protein L10
ambt_19650	12.16	11.17	10.93	9.80	50S Ribosomal protein L1
ambt_19655	12.51	10.98	11.98	10.65	50S Ribosomal protein L11
ambt_19860	10.52	10.18	10.26	10.57	RNA-Binding protein Hfq
ambt_19910	9.19	9.35	10.03	9.26	Anti-RNA polymerase sigma 70 factor
ambt_19930	11.58	11.65	11.14	11.87	OmpA/MotB protein
ambt_19980	11.75	9.46	11.83	10.54	30S Ribosomal protein S18
ambt_19985	11.94	11.08	12.37	11.15	30S Ribosomal protein S6
ambt_20300	12.63	12.47	14.78	13.88	Hypothetical protein
ambt_20305	9.91	9.32	10.47	10.41	Hypothetical protein
ambt_22160	11.87	10.35	9.17	9.50	F0F1 ATP synthase subunit alpha
ambt_22165	12.16	9.89	9.45	9.71	ATP synthase F1 subunit delta
ambt_22170	12.31	10.54	9.68	10.05	F0F1 ATP synthase subunit B
ambt_22175	12.03	10.32	9.15	9.58	F0F1 ATP synthase subunit C

Supplementary Table 2. Strain SN2 genes showing a more than two-fold differential expression (> $\log_2$ 2) in response to tidal flat conditions. TF, tidal flat; SW, seawater; P, pyruvate; N, naphthalene.

Gene	Differential gene expression (fold change)		Putative functions	COG category
	Log_2 (TF-N/SW-N)	Log_2 (TF-P/SW-P)		
ambt_00205	2.37	1.81	Elongation factor Tu	J
ambt_00630	1.28	1.22	Peptide methionine sulfoxide reductase MsrA	O
ambt_00655	2.79	2.22	Hypothetical protein	–
ambt_00680	1.20	1.11	Carboxylesterase	R
ambt_00715	4.05	2.69	Phage shock protein A	KT
ambt_00720	3.73	2.89	Phage shock protein C	KT
ambt_00725	3.73	2.46	Hypothetical protein	–
ambt_01150	2.10	1.19	Pyrroloquinoline quinone biosynthesis protein PqqE	R
ambt_01155	2.85	1.67	Pyrroloquinoline quinone biosynthesis protein PqqD	–
ambt_01160	1.11	1.01	Pyrroloquinoline quinone biosynthesis protein PqqC	H
ambt_01325	2.45	1.25	Hypothetical protein	–
ambt_01450	1.35	2.88	Hypothetical protein	–
ambt_01715	1.70	5.04	Putative AcnD-accessory protein PrpF	S
ambt_01720	2.14	6.74	Aconitate hydratase	C
ambt_01725	1.48	6.64	Methylcitrate synthase	C
ambt_02040	1.04	1.16	Twitching motility protein PilT	NU
ambt_02045	1.14	1.11	Twitching motility protein PilU (type IV pili)	NU
ambt_02460	1.42	2.59	Glycine cleavage system aminomethyltransferase T	E
ambt_02470	1.83	2.82	Glycine dehydrogenase	E
ambt_02550	2.92	2.37	Putative oxidoreductase	R
ambt_02670	2.92	1.34	Putative NADH oxidoreductase	CR
ambt_02850	2.04	1.38	Putative TonB-dependent receptor	P
ambt_03110	2.86	1.62	Lactoylglutathione lyase	E
ambt_03145	4.32	2.06	Hypothetical protein	–
ambt_03190	1.94	3.07	Acyl-CoA dehydrogenase	I
ambt_03320	3.13	2.11	TonB-dependent receptor	P
ambt_03325	3.67	3.38	Hypothetical protein	–
ambt_03330	3.14	2.27	PepSY-associated TM helix domain-containing protein	S
ambt_03335	2.48	1.12	Hypothetical protein	–
ambt_03350	4.14	1.45	TonB-dependent receptor	P
ambt_03715	1.62	2.41	Malate synthase G	C
ambt_03725	2.64	4.02	Isocitrate lyase	C
ambt_04070	3.44	1.25	ATP-dependent protease La	O
ambt_04185	3.62	1.20	Hypothetical protein	–
ambt_04305	1.54	1.20	Putative deoxyribonuclease	L
ambt_04365	2.32	1.16	3,4-Dihydroxy-2-butanone 4-phosphate synthase/GTP cyclohydrolase II	H
ambt_04470	2.26	2.20	ClpB protein	O
ambt_04585	5.01	3.18	Heat shock protein 90	O
ambt_05200	4.12	1.54	Ferrichrome-iron receptor	P
ambt_05265	1.21	1.04	Hypothetical protein	–
ambt_05520	1.25	1.53	Inosine/uridine-preferring nucleoside hydrolase	F
ambt_05990	1.36	1.41	Hypothetical protein	P
ambt_06935	1.47	1.23	Betaine aldehyde dehydrogenase	C
ambt_06940	1.26	1.01	Choline dehydrogenase	E
ambt_07685	1.79	2.11	Hypothetical protein	R
ambt_08480	1.50	1.26	Alanine dehydrogenase	E
ambt_08925	1.07	1.42	Acyl-CoA dehydrogenase	I
ambt_09130	2.20	3.09	Electron transfer flavoprotein subunit alpha	C
ambt_09135	2.07	3.61	Electron transfer flavoprotein subunit beta	C
ambt_09140	1.85	3.90	Electron transfer flavoprotein-ubiquinone oxidoreductase	C
ambt_09230	2.68	1.72	HlyD family secretion protein	M
ambt_09825	1.35	1.66	Short-chain dehydrogenase/reductase SDR	IQR
ambt_10270	1.93	5.11	Acetyl-CoA acetyltransferase with thiolase domain	I
ambt_10280	1.46	8.17	Isovaleryl-CoA dehydrogenase	I
ambt_11055	1.27	1.06	Succinylglutamate desuccinylase	E
ambt_11390	1.72	1.38	Hypothetical protein	–
ambt_11435	1.84	1.24	Small heat shock protein	O
ambt_11510	4.30	2.22	Chaperone protein DnaJ	O

ambt_11515	4.59	3.51	Molecular chaperone DnaK	O
ambt_11520	3.29	1.63	Heat shock protein GrpE	O
ambt_11595	2.79	1.14	Hypothetical protein	S
ambt_11920	2.65	1.09	Putative multidrug resistance protein	M
ambt_11935	1.30	1.29	Oxidoreductase, zinc-binding dehydrogenase family protein	CR
ambt_12260	1.88	1.88	O-Acetylhomoserine/O-acetylserine sulfhydrylase	E
ambt_12280	1.66	1.07	Citrate transporter	P
ambt_12365	1.27	2.00	Putative ATPase family protein	R
ambt_12370	1.73	1.97	Hypothetical protein	R
ambt_12375	2.12	2.45	Hypothetical protein	–
ambt_12380	2.02	2.37	Von Willebrand factor, type A	R
ambt_12390	1.34	1.01	batD protein	–
ambt_12395	1.64	1.76	Hypothetical protein	S
ambt_12540	1.12	1.03	Endonuclease III	L
ambt_12600	4.98	2.26	AhpC/Tsa family antioxidant	O
ambt_12785	1.06	2.11	Hypothetical protein	R
ambt_12965	2.12	1.69	TonB-dependent receptor	P
ambt_12970	5.05	3.84	OmpA-like protein	P
ambt_13015	2.97	1.45	Phage shock protein C	KT
ambt_13020	2.97	1.67	Phage shock protein B	–
ambt_13025	3.15	1.80	Phage shock protein A	KT
ambt_13340	1.41	1.26	N-acylneuraminate-9-phosphate synthase	M
ambt_13405	2.41	1.02	Hypothetical protein	–
ambt_13450	1.42	1.71	Flagellin-like protein	N
ambt_13780	3.12	1.24	Hypothetical protein	–
ambt_13875	1.04	1.54	Putative proton-dependent peptide transporter	E
ambt_14285	1.10	1.28	Putative transcriptional regulator OhrR	K
ambt_14405	1.70	1.09	S-adenosylmethionine synthetase	H
ambt_14610	1.94	1.52	Ribosomal subunit interface protein	J
ambt_14615	1.18	1.55	Nitrogen regulatory IIA protein	GT
ambt_14620	1.14	1.35	glmZ(sRNA)-inactivating NTPase	R
ambt_14625	1.60	1.63	Phosphocarrier protein NPR	G
ambt_14675	2.00	1.02	Ubiquinol-cytochrome c reductase, cytochrome B	C
ambt_14840	1.34	1.08	Bifunctional aconitate hydratase 2/2-methylisocitrate dehydratase	C
ambt_14870	5.21	1.21	Hypothetical protein	–
ambt_15350	1.10	2.13	ATP-dependent helicase HepA	KL
ambt_15525	1.57	1.70	Hypothetical protein	–
ambt_15715	1.02	1.32	Putative manganese transporter	P
ambt_15790	1.77	1.88	Putative dioxygenase	R
ambt_15795	1.20	2.37	NAD-dependent aldehyde dehydrogenase	C
ambt_15815	2.72	1.49	Thioredoxin domain-containing protein	O
ambt_15885	1.46	1.18	Delta-aminolevulinic acid dehydratase	H
ambt_16105	1.25	1.07	Sensory box/GGDEF family protein	T
ambt_16120	1.70	1.97	Hypothetical protein	ER
ambt_16130	1.50	1.07	Putative glutathione S-transferase	O
ambt_16240	1.06	1.18	Bifunctional N-succinyldiaminopimelate-aminotransferase/acetylornithine transaminase protein	E
ambt_16355	1.45	1.48	Hypothetical protein	E
ambt_16360	1.18	1.10	Hypothetical protein	–
ambt_16505	2.46	1.98	50S Ribosomal protein L16	J
ambt_16510	2.33	2.16	30S Ribosomal protein S3	J
ambt_16515	1.99	1.41	50S Ribosomal protein L22	J
ambt_16520	1.80	1.28	30S Ribosomal protein S19	J
ambt_16525	1.77	1.81	50S Ribosomal protein L2	J
ambt_16530	1.54	1.20	50S Ribosomal protein L23	J
ambt_16605	1.13	1.68	Twin-arginine translocation pathway signal protein	–
ambt_16610	1.65	1.74	Oxidoreductase	E
ambt_16620	2.06	1.13	Putative nucleoside permease	GEPR
ambt_16685	3.06	1.26	Major facilitator transporter	GEPR
ambt_16695	6.05	3.09	TonB-dependent receptor	P
ambt_16700	3.93	2.65	Hypothetical protein	–
ambt_17255	1.90	1.95	Putative carbohydrate kinase	C
ambt_17890	1.16	1.44	Aromatic-ring-hydroxylating dioxygenase subunit beta	Q
ambt_17895	1.35	1.57	Ring hydroxylating dioxygenase subunit alpha	PR

ambt_17905	1.04	1.01	Hypothetical protein	–
ambt_17910	1.34	1.05	Dihydrodipicolinate synthetase	EM
ambt_17915	1.66	1.33	Aldehyde dehydrogenase	C
ambt_17955	1.42	1.16	Filamentation induced by cAMP protein fic	S
ambt_17980	1.61	1.90	Fumarylacetoacetate (FAA) hydrolase	Q
ambt_17990	1.10	1.78	Maleylacetoacetate isomerase	O
ambt_18150	1.03	2.58	Hypothetical protein	–
ambt_18240	2.60	1.76	Porin family outer membrane protein	–
ambt_18485	1.92	1.65	Hypothetical protein	S
ambt_18490	1.91	1.52	Hypothetical protein	R
ambt_18545	1.77	3.58	Transcriptional regulatory protein	K
ambt_18550	4.19	3.52	FhuE receptor	P
ambt_18600	1.14	1.23	Helix-turn-helix domain-containing protein	K
ambt_19020	1.62	1.11	Hypothetical protein	M
ambt_19085	1.85	1.29	30S Ribosomal protein S5	J
ambt_19135	1.29	1.63	Cold-active serine alkaline protease	O
ambt_19260	2.71	2.02	3-Methyl-adenine DNA glycosylase	L
ambt_19265	2.44	2.66	Oligopeptidase A	E
ambt_19340	3.69	2.45	ATP-Dependent protease ATP-binding subunit HslU	O
ambt_19345	3.51	3.27	ATP-Dependent protease subunit HslV	O
ambt_19470	3.16	1.59	Aminopeptidase B	E
ambt_19475	2.88	2.80	Hypothetical protein	E
ambt_19550	4.98	3.70	Putative outer membrane receptor for iron transport	P
ambt_19610	2.01	1.54	Elongation factor Tu	J
ambt_19630	1.03	1.54	DNA-directed RNA polymerase subunit beta'	K
ambt_19640	3.30	1.09	50S Ribosomal protein L7/L12	J
ambt_19650	1.23	1.37	50S Ribosomal protein L1	J
ambt_19785	1.59	2.02	homocysteine S-methyltransferase family protein	E
ambt_20030	2.05	2.25	Hypothetical protein	–
ambt_20035	2.71	2.57	Hypothetical protein	P
ambt_20040	2.33	2.16	Hemin ABC transporter ATPase	P
ambt_20045	1.65	2.34	Hemin ABC transporter pemease component	P
ambt_20050	3.65	4.29	Hemin ABC transporter periplasmic protein	P
ambt_20055	4.08	4.36	Hypothetical protein	–
ambt_20060	4.19	3.55	Ferrienterochelin/colicin outer membrane receptor	P
ambt_20100	1.10	2.01	Hypothetical protein	–
ambt_20225	2.37	1.27	Hypothetical protein	–
ambt_20285	2.15	3.29	Hypothetical protein	–
ambt_20290	1.94	4.60	Outer membrane protein	–
ambt_20295	1.72	3.87	RND superfamily exporter	R
ambt_20375	1.95	5.59	CBS domain-containing protein	R
ambt_20420	1.99	1.15	Long-chain fatty acid transport protein	I
ambt_20430	2.37	1.75	Putative NADP-dependent oxidoreductase	R
ambt_20640	1.07	1.20	Putative urease subunit beta	E
ambt_20850	1.27	1.43	Hypothetical protein	–
ambt_20945	2.27	2.19	Hypothetical protein	–
ambt_20950	1.53	2.01	Lytic transglycosylase, catalytic	M
ambt_21005	3.25	2.35	Integrase family protein	L
ambt_21010	5.29	3.70	Chaperonin GroEL	O
ambt_21015	5.16	2.68	Co-chaperonin GroES	O
ambt_21485	2.31	1.01	Porin family outer membrane protein	–
ambt_21540	1.92	1.04	Transcription elongation factor GreB	K
ambt_21920	1.48	1.63	Hypothetical protein	–
ambt_22030	1.25	1.82	Hypothetical protein	R
ambt_22035	1.38	2.45	Hypothetical protein	K
ambt_22040	1.87	2.16	Hypothetical protein	R
ambt_22050	2.57	1.10	HipA domain-containing protein	R
ambt_22150	3.30	1.79	F0F1 ATP synthase subunit beta	C

Supplementary Table 3. Strain SN2 genes showing a more than two-fold differential expression (> $\log_2$ 2) in response to seawater conditions. TF, tidal flat; SW, seawater; P, pyruvate; N, naphthalene.

Gene	Differential gene expression (fold change)		Putative functions	COG category
	Log_2 (SW-N/TF-N)	Log_2 (SW-P/TF-P)		
ambt_00005	2.95	4.07	Chromosomal replication initiator protein dnaA	L
ambt_00010	1.05	1.41	DNA polymerase III subunit beta	L
ambt_00045	1.23	1.03	Hypothetical protein	–
ambt_00050	1.26	2.23	Hypothetical protein	S
ambt_00080	4.49	3.26	TraT complement resistance	–
ambt_00085	4.27	2.80	Hypothetical protein	–
ambt_00140	2.44	1.65	Outer membrane protein OmpW	M
ambt_00145	1.92	1.07	Putative hemoglobin-like oxygen-binding protein	R
ambt_00180	3.58	2.96	Hypothetical protein	–
ambt_00220	1.15	1.56	Hypothetical protein	–
ambt_00250	1.09	1.70	Ankyrin	R
ambt_00265	1.36	5.51	nptA protein	P
ambt_00405	1.32	1.74	Hypothetical protein	–
ambt_00540	1.79	4.87	PAS sensor diguanylate cyclase and phosphodiesterase	T
ambt_00570	2.43	2.69	Hypothetical protein	–
ambt_00600	1.04	2.07	Biopolymer transport protein ExbD/TolR	U
ambt_00635	2.82	1.19	Putative redox protein	S
ambt_00740	2.07	1.57	TonB-dependent receptor, plug	–
ambt_00745	2.50	1.27	Sulfite reductase subunit alpha	P
ambt_00785	2.14	1.85	Putative delta-9 fatty acid desaturase	I
ambt_00825	5.39	3.29	Hypothetical protein	TZD R
ambt_00830	1.05	1.23	DNA polymerase II	L
ambt_00855	5.54	3.62	Putative aminotransferase	E
ambt_00860	6.20	4.09	Membrane protein	R
ambt_00865	7.22	4.58	TonB-dependent receptor	P
ambt_00875	2.91	2.06	Endonuclease/exonuclease/phosphatase	R
ambt_00880	2.76	2.18	phospholipase D/transphosphatidylase	I
ambt_00890	1.53	2.44	potassium or sodium transport protein TrkB	P
ambt_00955	4.06	2.32	ATP-dependent RNA helicase RhlE	LKJ
ambt_00960	1.87	2.52	FHA modulated ABC efflux pump with fused ATPase and integral membrane subunits	V
ambt_00965	1.91	2.28	Zinc metalloprotease	E
ambt_00970	3.78	4.56	protein serine/threonine phosphatase	T
ambt_00975	3.49	2.30	Serine/threonine protein kinase	RTK L
ambt_00980	2.86	2.91	Hypothetical protein	–
ambt_00985	2.97	2.14	Hypothetical protein	NT
ambt_00990	2.65	2.93	Hypothetical protein	T
ambt_00995	2.56	2.60	Hypothetical protein	U
ambt_01000	2.54	1.42	Hypothetical protein	L
ambt_01005	2.92	2.24	Serine/threonine protein phosphatase, putative	T
ambt_01010	3.17	3.23	Forkhead-associated protein	O
ambt_01015	2.26	3.13	Hypothetical protein	–
ambt_01020	1.35	2.83	Hypothetical protein	–
ambt_01265	2.07	1.38	Hypothetical protein	R
ambt_01270	2.61	2.08	Igma-70 family RNA polymerase sigma factor	K
ambt_01275	1.62	2.03	Hypothetical protein	–
ambt_01280	1.59	1.80	Hypothetical protein	–
ambt_01335	1.09	3.18	Lipase-like protein	I
ambt_01395	1.25	2.11	RDD domain-containing protein	S
ambt_01500	1.91	1.10	Class I and II aminotransferase	E
ambt_01640	1.56	1.33	Hypothetical protein	–
ambt_01645	1.03	2.55	Putative phage shock protein E	P
ambt_01710	1.11	1.52	Response regulator containing a CheY-like receiver domain and a GGDEF domain	T

ambt_01790	3.08	1.28	Hypothetical protein	–
ambt_01920	1.74	1.00	Hypothetical protein	–
ambt_01970	4.98	3.64	Hypothetical protein	–
ambt_01975	1.26	1.79	Glucose-6-phosphate isomerase	G
ambt_02005	1.45	1.79	Glyoxalase/bleomycin resistance protein/dioxygenase	E
ambt_02075	4.63	5.33	Hypothetical protein	–
ambt_02080	2.35	2.16	Mechanosensitive ion channel MscS	M
ambt_02485	1.53	2.27	Hypothetical protein	–
ambt_02520	9.30	2.23	Hypothetical protein	–
ambt_02530	1.09	1.51	MATE efflux family protein	V
ambt_02535	4.68	2.64	Hypothetical protein	–
ambt_02540	3.56	4.19	Hypothetical protein	–
ambt_02545	1.75	1.99	Peptidase S9, prolyl oligopeptidase active site region	E
ambt_02570	4.43	1.44	PEP motif-containing protein	–
ambt_02575	2.80	1.05	Hypothetical protein	–
ambt_02580	2.21	1.47	Hypothetical protein	–
ambt_02585	2.88	1.26	Hypothetical protein	–
ambt_02640	1.48	1.49	Putative kinase	TD
ambt_02680	1.72	3.04	SecC motif-containing protein	–
ambt_02685	2.81	3.06	Alpha amylase catalytic subunit	G
ambt_02695	2.09	1.76	Hypothetical protein	–
ambt_02700	2.93	1.84	Hypothetical protein	–
ambt_02705	3.95	2.65	Hypothetical protein	C
ambt_02710	4.57	3.56	CBS domain-containing protein	R
ambt_02720	1.25	1.62	Family 2 glycosyl transferase	M
ambt_02740	1.96	1.21	Lysine exporter protein LysE/YggA	E
ambt_02750	2.48	1.99	Late embryogenesis abundant protein	–
ambt_02780	2.85	1.58	Heat shock protein DnaJ-like protein	O
ambt_02795	1.21	3.55	Hypothetical protein	–
ambt_02800	1.20	4.89	Glycosyl transferase family protein	M
ambt_02865	2.92	1.95	Hypothetical protein	O
ambt_02870	2.65	2.13	Protease	R
ambt_02875	2.19	2.24	Short-chain alcohol dehydrogenase	R
ambt_02880	3.33	2.54	Hypothetical protein	S
ambt_02945	1.75	6.52	Metallophosphoesterase	–
ambt_02965	4.33	2.30	Hypothetical protein	–
ambt_02970	3.12	3.15	Hypothetical protein	O
ambt_02975	1.61	1.39	Hypothetical protein	T
ambt_03000	1.64	1.16	Hypothetical protein	–
ambt_03005	5.84	1.24	Twin-arginine translocation pathway signal	E
ambt_03035	3.32	2.10	Hypothetical protein	M
ambt_03040	3.30	1.75	Hypothetical protein	–
ambt_03045	1.76	1.37	Hypothetical protein	–
ambt_03135	1.67	1.15	Hypothetical protein	S
ambt_03165	4.38	1.03	TonB-dependent receptor	P
ambt_03170	4.87	3.18	Peptidase M19, renal dipeptidase	E
ambt_03265	1.38	4.19	Hypothetical protein	HJ
ambt_03360	1.12	1.47	Hypothetical protein	S
ambt_03395	1.23	1.10	Hypothetical protein	–
ambt_03430	2.79	4.19	Hypothetical protein	–
ambt_03435	1.90	2.26	Hypothetical protein	–
ambt_03445	4.01	1.29	Helix-turn-helix domain-containing protein	K
ambt_03535	1.03	1.16	Putative URI domain endonuclease	L
ambt_03565	1.39	1.89	Polysaccharide biosynthesis/export protein	M
ambt_03595	1.78	1.33	Hypothetical protein	M
ambt_03665	1.15	1.33	Acetyltransferase	E
ambt_03670	1.81	2.45	Hypothetical protein	R
ambt_03690	1.18	1.02	Hypothetical protein	S
ambt_03700	1.07	1.87	Hypothetical protein	–
ambt_03705	1.29	1.52	Capsular polysaccharide biosynthesis protein I	MG
ambt_03735	1.11	1.40	Alkaline phosphatase	P
ambt_03820	4.64	3.58	TonB-dependent receptor, plug	P

ambt_03825	3.84	2.63	Hypothetical protein	–
ambt_03830	2.63	1.81	Diguanylate cyclase/phosphodiesterase	T
ambt_03875	1.78	2.81	Hypothetical protein	–
ambt_03890	1.07	1.85	Hypothetical protein	H
ambt_03990	1.31	2.07	GTP-binding protein Der	R
ambt_03995	1.56	1.72	Hypothetical protein	–
ambt_04085	2.51	5.44	Succinyl-diaminopimelate desuccinylase	E
ambt_04090	1.18	5.00	Acyltransferase family protein	R
ambt_04095	1.94	7.74	Metallophosphoesterase	S
ambt_04195	1.75	1.05	Putative prepilin peptidase dependent protein	NU
ambt_04255	3.11	1.87	RNA polymerase sigma factor RpoE	K
ambt_04260	3.19	1.18	Sigma E factor negative regulatory protein	T
ambt_04265	2.11	1.43	Putative anti sigma E (sigma 24) factor	T
ambt_04270	1.90	1.01	Sigma-E factor regulatory protein RseC	T
ambt_04275	1.13	2.21	Hypothetical protein	–
ambt_04440	1.70	2.47	Hypothetical protein	R
ambt_04445	1.65	1.53	RNA polymerase sigma factor	K
ambt_04450	1.74	1.03	Transcription negative regulator ChrR	T
ambt_04455	1.23	1.50	Competence lipoprotein Coml	R
ambt_04460	1.17	1.36	Ribosomal large subunit pseudouridine synthase D	J
ambt_04480	1.76	3.26	Hypothetical protein	–
ambt_04490	1.76	1.86	CDP-diacylglycerol--serine O-phosphatidyltransferase	I
ambt_04500	4.61	2.31	Hypothetical protein	–
ambt_04510	2.19	1.47	Hypothetical protein	–
ambt_04515	1.03	1.34	Hypothetical protein	–
ambt_04530	1.23	2.09	Hypothetical protein	–
ambt_04560	2.73	6.48	Vanadium-dependent bromoperoxidase 2	–
ambt_04680	3.26	1.79	Hypothetical protein	–
ambt_04685	3.87	3.76	Hypothetical protein	–
ambt_04725	3.58	1.97	Hypothetical protein	R
ambt_04820	2.92	1.66	Hypothetical protein	R
ambt_04925	5.60	1.17	Hypothetical protein	S
ambt_04960	1.36	2.31	Hypothetical protein	GER
ambt_04965	1.59	2.52	Putative fatty acid desaturase	I
ambt_04970	1.43	2.39	Putative Oxidoreductase	C
ambt_05090	1.20	1.21	Hypothetical protein	–
ambt_05145	1.60	3.50	Hypothetical protein	–
ambt_05170	2.60	6.89	Hypothetical protein	–
ambt_05175	3.25	7.06	Alkaline phosphatase	P
ambt_05180	1.95	6.61	Alkaline phosphatase	P
ambt_05250	2.22	2.82	Hypothetical protein	–
ambt_05280	1.54	1.16	Hypothetical protein	–
ambt_05405	1.78	1.86	Hypothetical protein	–
ambt_05410	1.22	1.28	Hypothetical protein	–
ambt_05415	1.75	2.88	Hypothetical protein	–
ambt_05420	1.80	1.88	Hypothetical protein	–
ambt_05425	1.40	1.61	Hypothetical protein	S
ambt_05465	1.97	1.18	Hypothetical protein	R
ambt_05470	1.47	1.41	Hypothetical protein	–
ambt_05575	2.53	2.19	Superoxide dismutase, copper/zinc binding protein	P
ambt_05585	2.25	3.14	Osmotically inducible protein	O
ambt_05615	1.43	1.64	Putative diguanylate cyclase/phosphodiesterase	T
ambt_05705	2.45	4.06	Extracellular ribonuclease/nuclease fusion protein	L
ambt_05710	2.11	2.32	Hypothetical protein	–
ambt_05735	1.56	1.87	Family 2 glycosyl transferase	M
ambt_05765	1.51	1.86	Hypothetical protein	–
ambt_05770	1.76	2.08	Hypothetical protein	–
ambt_05785	3.01	2.11	Hypothetical protein	R
ambt_05790	2.78	2.68	Hypothetical protein	–
ambt_05915	3.99	3.38	Hypothetical protein	–
ambt_05920	4.83	3.56	Hypothetical protein	–
ambt_05935	2.28	1.52	Hypothetical protein	–

ambt_05940	3.13	2.52	Hypothetical protein	–
ambt_05945	3.82	3.01	Hypothetical protein	–
ambt_05975	1.98	2.73	Putative exopolysaccharide synthesis protein	R
ambt_05980	1.20	2.39	Hypothetical protein	–
ambt_06010	1.70	2.42	Hypothetical protein	–
ambt_06080	1.56	2.83	Hypothetical protein	E
ambt_06085	4.18	2.57	histidinol-phosphate aminotransferase	E
ambt_06195	1.10	1.30	Hypothetical protein	S
ambt_06205	1.53	1.35	Hypothetical protein	–
ambt_06230	1.90	2.12	Hypothetical protein	–
ambt_06235	1.76	2.18	Hypothetical protein	–
ambt_06375	1.43	2.00	Hypothetical protein	–
ambt_06395	2.58	2.65	Hypothetical protein	–
ambt_06400	1.66	1.02	alpha/beta hydrolase fold-domain containing protein	R
ambt_06405	1.77	1.92	Putative transposase protein, Y4bF	L
ambt_06505	5.74	1.32	coproporphyrinogen III oxidase	H
ambt_06510	2.76	2.05	Hypothetical protein	H
ambt_06590	1.85	1.07	alpha/beta hydrolase fold protein	R
ambt_06645	1.72	1.57	Hypothetical protein	–
ambt_06650	1.03	2.47	Hypothetical protein	–
ambt_06680	1.39	2.66	Hypothetical protein	S
ambt_06710	2.28	1.02	Hypothetical protein	S
ambt_06720	2.51	2.06	diguanylate cyclase	T
ambt_06725	2.35	1.07	alpha/beta hydrolase fold protein	R
ambt_06740	1.31	2.38	2OG-Fe(II) oxygenase family oxidoreductase	O
ambt_06750	1.45	3.50	Putative extracellular nuclease	R
ambt_06790	1.08	1.22	cupin 2 barrel domain-containing protein	Q
ambt_06795	1.58	1.65	Hypothetical protein	C
ambt_06800	1.17	2.00	Hypothetical protein	–
ambt_06965	1.07	1.32	Hypothetical protein	K
ambt_06995	1.11	1.06	Hypothetical protein	L
ambt_07005	1.09	2.63	Hypothetical protein	–
ambt_07050	2.57	1.51	TonB-dependent receptor	P
ambt_07090	4.83	1.05	F0F1-ATPase subunit, putative	–
ambt_07100	4.98	1.88	F0F1 ATP synthase subunit beta	C
ambt_07120	2.75	1.59	Hypothetical protein	–
ambt_07395	1.28	1.41	Hypothetical protein	–
ambt_07640	1.58	1.90	Putative cold-shock RNA methyltransferase	J
ambt_07645	1.02	1.08	Hypothetical protein	–
ambt_07885	3.20	1.56	Hypothetical protein	–
ambt_07895	2.97	1.45	Hypothetical protein	–
ambt_08105	1.71	1.05	3-Hydroxydecanoyl-(acyl carrier protein) dehydratase	I
ambt_08140	2.06	1.08	Hypothetical protein	–
ambt_08185	3.50	1.12	Hypothetical protein	C
ambt_08250	1.41	1.20	Hypothetical protein	S
ambt_08270	1.43	1.39	Transcriptional regulator	T
ambt_08365	1.93	3.58	Hypothetical protein	–
ambt_08375	1.05	2.50	Hypothetical protein	MG
ambt_08540	1.03	1.44	Ribonucleotide-diphosphate reductase subunit alpha	F
ambt_08550	1.18	1.86	Iron-sulfur cluster-binding protein	C
ambt_08600	1.60	1.02	Hypothetical protein	–
ambt_08610	2.04	1.14	Hypothetical protein	–
ambt_08625	1.20	1.38	Hypothetical protein	–
ambt_08640	1.56	1.09	Hypothetical protein	–
ambt_08645	1.87	1.46	Hypothetical protein	–
ambt_08665	1.81	1.08	Hypothetical protein	–
ambt_08675	1.51	2.05	Hypothetical protein	–
ambt_08695	1.22	1.26	RNA-directed DNA polymerase	L
ambt_08700	1.55	1.58	Putative inner membrane protein	–
ambt_08740	1.64	1.34	Hypothetical protein	–
ambt_08745	1.63	1.94	Hypothetical protein	–
ambt_08785	1.68	1.28	Hypothetical protein	–

ambt_09210	2.55	1.00	Allophanate hydrolase subunit 1	E
ambt_09245	6.27	4.98	Hypothetical protein	–
ambt_09265	2.34	2.43	DNA polymerase 3 subunit epsilon	MG
ambt_09270	1.31	3.11	Hypothetical protein	–
ambt_09275	1.87	2.18	Cytochrome c oxidase subunit II	C
ambt_09280	1.42	1.20	Cytochrome-c oxidase	C
ambt_09285	1.22	1.03	Cytochrome c oxidase subunit III	C
ambt_09290	1.05	1.07	Hypothetical protein	–
ambt_09295	1.50	1.27	Cytochrome c oxidase caa3-type, assembly factor CtaG-like protein	S
ambt_09345	6.37	7.67	Hypothetical protein	–
ambt_09350	5.40	6.08	5-Methyltetrahydropteroyltriglutamate-- homocysteine methyltransferase	E
ambt_09355	1.47	2.50	Hypothetical protein	–
ambt_09405	2.24	1.55	Restriction endonuclease	V
ambt_09410	1.63	1.40	Mechanosensitive ion channel MscS	M
ambt_09435	1.04	1.47	DNA topoisomerase	L
ambt_09440	2.67	2.45	Hypothetical protein	–
ambt_09445	1.26	2.25	Hypothetical protein	–
ambt_09450	3.05	1.14	Hypothetical protein	I
ambt_09455	4.12	2.20	Hypothetical protein	–
ambt_09460	2.09	1.88	Hypothetical protein	S
ambt_09645	1.15	2.29	Translation initiation factor IF-1	J
ambt_09665	3.21	1.77	Stress response protein CspD	K
ambt_09770	1.19	1.23	Hypothetical protein	–
ambt_09895	1.97	1.63	Esterase	R
ambt_09935	3.74	3.29	GGDEF domain-containing protein	T
ambt_10100	1.08	1.65	Transposase	–
ambt_10205	2.38	4.47	AraC family transcriptional regulator	K
ambt_10210	2.68	6.14	GGDEF domain-containing protein	T
ambt_10215	3.28	8.32	Putative tonB-dependent receptor	P
ambt_10220	2.99	3.97	Acid phosphatase	R
ambt_10225	2.40	4.39	Putative phytase domain-containing protein	I
ambt_10320	2.25	1.75	Permease of the major facilitator superfamily protein	–
ambt_10485	2.71	1.22	Hypothetical protein	–
ambt_10490	1.73	1.69	Hypothetical protein	–
ambt_10495	1.09	1.11	Bactoprenol glucosyl transferase	M
ambt_10500	1.62	1.99	Bactoprenol-linked glucose translocase	S
ambt_10525	1.05	1.03	Hypothetical protein	–
ambt_10565	1.05	1.36	Transposase	–
ambt_10590	1.85	1.68	Dolichyl-phosphate mannose synthase-like protein	M
ambt_10595	1.75	1.22	Glycosyl transferase family protein	M
ambt_10600	1.61	1.77	Hypothetical protein	–
ambt_10605	1.84	1.81	Glycosyltransferase	M
ambt_10610	2.51	2.64	Serine O-acetyltransferase	E
ambt_10615	1.93	2.39	Hypothetical protein	–
ambt_10620	2.55	2.16	Putative glycosyltransferase	M
ambt_10635	1.70	1.32	Hypothetical protein	–
ambt_10660	2.37	2.63	Putative Exopolysaccharide biosynthesis protein	M
ambt_10665	3.17	2.65	polysaccharide export periplasmic protein	M
ambt_10670	2.82	1.79	Hypothetical protein	–
ambt_10735	1.76	1.79	Amidase	J
ambt_10740	1.73	1.25	Gamma-glutamyltransferase	E
ambt_10745	1.68	1.41	Hypothetical protein	R
ambt_10750	2.07	1.09	Class V aminotransferase	E
ambt_10755	2.40	1.49	Allantoate amidohydrolase	E
ambt_10760	3.65	2.19	Hypothetical protein	S
ambt_10765	1.68	1.09	Hypothetical protein	–
ambt_10780	6.47	1.89	TonB-dependent receptor	P
ambt_10785	4.51	1.25	Purine nucleoside permease	F
ambt_10790	4.16	3.12	Signal transduction histidine kinase	T
ambt_10800	1.96	1.20	Response regulator receiver modulated diguanylate cyclase/phosphodiesterase with PAS/PAC sensor(s)	T

ambt_10815	3.11	1.67	Xanthine/uracil/vitamin C permease	R
ambt_11015	1.43	3.39	Amino acid-binding ACT domain-containing protein	S
ambt_11020	1.56	2.39	Hypothetical protein	G
ambt_11065	3.02	1.95	Starch phosphorylase	G
ambt_11070	1.47	1.15	Phosphoglucomutase	G
ambt_11095	10.22	4.85	Formate/nitrite transporter	P
ambt_11100	9.53	3.92	Protein serine/threonine phosphatase	T
ambt_11105	6.95	5.77	Cyanate hydratase	P
ambt_11250	1.10	1.17	Transferase hexapeptide repeat containing protein	E
ambt_11295	2.18	1.63	N-Acetyl-mannosamine transferase	M
ambt_11300	2.88	2.72	Family 2 glycosyl transferase	M
ambt_11305	2.54	2.18	Glycosyl transferase family protein	M
ambt_11310	2.86	1.76	Family 2 glycosyl transferase	M
ambt_11315	2.43	1.74	Polysaccharide biosynthesis protein	R
ambt_11320	3.18	1.77	Hypothetical protein	L
ambt_11330	1.22	1.13	Putative exopolysaccharide biosynthesis protein	M
ambt_11335	1.64	1.52	Polysaccharide export protein	M
ambt_11340	1.87	1.81	Hypothetical protein	–
ambt_11345	1.42	1.73	Transglutaminase family protein cysteine peptidase BTLCP	S
ambt_11350	4.19	2.08	Hypothetical protein	–
ambt_11355	3.16	1.55	Hypothetical protein	–
ambt_11375	2.10	1.55	Putative alkylhydroperoxidase AhpD family protein	S
ambt_11440	1.14	1.26	Hypothetical protein	–
ambt_11500	2.53	1.59	Sensory box/GGDEF/EAL domain-containing protein	T
ambt_11565	1.72	1.41	Hypothetical protein	–
ambt_11640	1.42	1.51	PHP domain-containing protein	R
ambt_11645	2.70	1.35	Anthranilate synthase component I	EH
ambt_11680	2.82	2.85	General stress protein	R
ambt_11725	1.26	1.42	Lipase/acylhydrolase, GDSL family protein	E
ambt_11735	1.95	1.07	Hypothetical protein	–
ambt_11775	1.34	1.19	Molybdenum cofactor biosynthesis protein A	H
ambt_11780	1.06	1.49	ABC transporter ATP-binding protein	O
ambt_11850	2.93	1.32	Hypothetical protein	–
ambt_11860	1.51	1.49	Glutathione synthase	HJ
ambt_11950	2.79	2.68	Cytochrome b561	C
ambt_11955	2.02	3.43	Catalase	P
ambt_12040	1.50	1.93	Hypothetical protein	–
ambt_12045	1.92	1.36	Methionine aminopeptidase	J
ambt_12145	1.37	1.38	Putative GAF sensor protein	T
ambt_12220	1.16	1.02	Putative esterase	R
ambt_12575	2.41	3.16	Hypothetical protein	K
ambt_12675	3.47	1.18	Smr protein/MutS2	S
ambt_12680	4.38	1.58	Hypothetical protein	–
ambt_12840	3.99	1.09	LysR family transcriptional regulator	K
ambt_12845	2.21	1.10	Hypothetical protein	GER
ambt_12855	1.33	1.59	Hypothetical protein	–
ambt_12990	1.07	1.00	AsnC family transcriptional regulator	K
ambt_13075	3.32	3.16	Hypothetical protein	–
ambt_13115	1.26	3.09	TonB-dependent receptor	P
ambt_13125	1.75	4.85	Carbonic anhydrase	P
ambt_13255	1.22	1.09	Flagellar motor switch protein FliM	N
ambt_13540	3.00	1.52	Hypothetical protein	S
ambt_13550	1.18	1.39	Hypothetical protein	S
ambt_13670	1.01	1.35	Membrane-associated zinc metalloprotease	M
ambt_13745	1.04	2.71	Hypothetical protein	–
ambt_13750	5.35	5.99	Hypothetical protein	–
ambt_13755	5.90	5.34	Hypothetical protein	–
ambt_13830	2.47	1.31	Putative glucose-1-phosphate uridylyltransferase	M
ambt_13845	1.22	2.03	UDP-phosphate alpha-N-acetylglucosaminyl 1-phosphate transferase	M
ambt_14000	1.35	1.77	2-Isopropylmalate synthase	E
ambt_14095	4.35	4.03	Hypothetical protein	–

ambt_14115	2.12	1.16	Hypothetical protein	–
ambt_14140	1.90	2.38	Hypothetical protein	L
ambt_14170	1.25	1.06	Hypothetical protein	K
ambt_14210	3.43	1.88	Hypothetical protein	–
ambt_14215	3.32	1.20	Hypothetical protein	–
ambt_14220	1.20	2.07	ABC transporter ATP-binding protein	R
ambt_14260	3.21	3.45	Pas/Pac sensor containing methyl-accepting chemotaxis sensory transducer	T
ambt_14340	1.21	1.37	Universal stress protein UspA	T
ambt_14470	2.71	1.33	Hypothetical protein	–
ambt_14655	2.04	1.39	Hypothetical protein	–
ambt_14970	1.05	1.50	UDP pyrophosphate phosphatase	V
ambt_15110	1.39	3.35	TonB-dependent receptor	H
ambt_15120	2.37	2.12	Putative hydrolase	R
ambt_15125	9.93	7.02	Hypothetical protein	–
ambt_15130	9.53	5.50	Nitrate transport ATP-binding subunits C and D	P
ambt_15135	11.55	5.50	Putative nitrate ABC transporter permease	P
ambt_15140	11.44	5.84	Nitrate/sulfonate/bicarbonate ABC transporter periplasmic protein	P
ambt_15145	6.61	4.92	Hypothetical protein	–
ambt_15155	3.01	3.86	Hypothetical protein	E
ambt_15160	6.05	5.25	Hypothetical protein	H
ambt_15165	6.60	5.44	Hypothetical protein	–
ambt_15170	6.21	3.11	Nitrate reductase	C
ambt_15175	7.87	5.43	Nitrite reductase (NAD(P)H) small subunit	PR
ambt_15180	9.78	5.63	Nitrite reductase	C
ambt_15190	1.25	1.81	Hypothetical protein	–
ambt_15195	6.63	1.79	Nitrate-and nitrite-responsive positive regulator	T
ambt_15215	1.10	1.02	Alkylated DNA repair protein	L
ambt_15330	1.38	1.40	ATP-dependent RNA helicase	LKJ
ambt_15400	1.33	1.50	Hypothetical protein	R
ambt_15510	3.87	3.41	Putative lipoprotein NlpD	M
ambt_15515	4.09	2.81	Sigma S (sigma 38) factor of RNA polymerase, major sigma factor during stationary phase	K
ambt_15645	1.40	1.35	Hypothetical protein	C
ambt_15655	1.26	2.05	Hypothetical protein	R
ambt_15680	1.31	1.43	Acetyltransferase	J
ambt_15720	1.46	1.12	Hypothetical protein	–
ambt_15745	2.53	1.17	Hypothetical protein	S
ambt_15825	2.30	1.21	Glucose dehydrogenase	G
ambt_15860	1.49	2.01	Peptidase M20D, amidohydrolase	R
ambt_15975	1.55	2.41	Phosphatidylserine decarboxylase	I
ambt_15990	1.05	2.61	MerC-like membrane protein	–
ambt_16060	2.77	1.06	Flagellar L-ring protein	N
ambt_16065	3.12	1.70	Hypothetical protein	–
ambt_16075	2.53	1.08	N-Acetyl-gamma-glutamyl-phosphate reductase	E
ambt_16135	1.29	4.03	Hypothetical protein	–
ambt_16210	7.33	1.05	Hypothetical protein	–
ambt_16330	7.61	1.11	Nitrogen regulatory protein P-II	E
ambt_16465	2.60	3.98	Hypothetical protein	–
ambt_16485	2.51	3.99	Lipopolysaccharide heptosyltransferase-1	M
ambt_16490	1.37	2.05	Glycoside hydrolase family protein	M
ambt_16635	1.75	1.36	LacI family transcriptional repressor	K
ambt_16660	1.98	1.89	Putative two-component system sensor kinase/response regulator fusion protein	T
ambt_16805	2.60	1.55	Hypothetical protein	–
ambt_16905	1.30	1.09	Filamentation induced by cAMP protein Fic	S
ambt_16930	2.64	4.22	Hypothetical protein	–
ambt_17000	2.27	1.22	Hypothetical protein	–
ambt_17365	1.78	1.16	Hypothetical protein	–
ambt_17405	1.72	1.12	Excinuclease ABC subunit A	L
ambt_17460	3.89	7.39	PA-phosphatase-like phosphoesterase	I
ambt_17465	2.99	6.33	Glycosyl transferase family protein	GC

ambt_17470	1.33	2.79	Hypothetical protein	–
ambt_17610	2.25	1.02	RES domain-containing protein	S
ambt_17615	1.38	1.03	Hypothetical protein	–
ambt_17740	2.40	2.12	Hypothetical protein	–
ambt_18245	2.02	2.21	Mechanosensitive ion channel family protein	M
ambt_18355	1.97	1.69	Phenylhydantoinase	F
ambt_18360	2.47	1.36	NCS1 nucleoside transporter	FH
ambt_18365	3.66	1.21	Amidase, hydantoinase/carbamoylase family protein	E
ambt_18390	4.77	1.70	FO synthase subunit 1	HR
ambt_18395	3.27	1.62	Hypothetical protein	HR
ambt_18400	2.91	1.32	LPPG:FO 2-phospho-L-lactate transferase	S
ambt_18405	3.78	1.47	Hypothetical protein	S
ambt_18415	1.82	1.37	NADPH-dependent F420 reductase	R
ambt_18445	4.11	1.46	Thiamine biosynthesis protein ThiC	H
ambt_18450	4.04	1.23	Thiamine monophosphate synthase	H
ambt_18455	3.75	1.98	Thiamine biosynthesis protein ThiF	H
ambt_18470	3.39	1.20	Thiamine biosynthesis protein ThiH	HR
ambt_18530	3.24	2.33	Putative membrane fusion protein	M
ambt_18610	1.32	1.09	Hypothetical protein	–
ambt_18725	1.60	1.43	CDP-glycerol:poly(glycerophosphate) glycerophosphotransferase	M
ambt_18745	1.60	2.14	Hypothetical protein	–
ambt_18755	1.67	1.64	DNA repair protein RadC	L
ambt_18825	2.15	1.51	Hypothetical protein	–
ambt_18855	2.02	1.97	Hypothetical protein	Q
ambt_18865	1.50	1.12	Nucleotide sugar dehydrogenase	M
ambt_18895	2.73	2.28	Hypothetical protein	–
ambt_18910	4.22	3.42	Hypothetical protein	–
ambt_18985	1.86	1.08	Transposase	–
ambt_19000	1.77	1.62	Hypothetical protein	–
ambt_19125	3.81	2.70	GAF sensor hybrid histidine kinase	T
ambt_19150	2.19	3.89	Phosphodiesterase/alkaline phosphatase D	P
ambt_19155	1.86	5.82	Outer membrane protein	P
ambt_19270	2.08	1.01	Response regulator	T
ambt_19280	3.00	1.17	Signal transduction histidine kinase	T
ambt_19360	2.75	1.38	Hypothetical protein	–
ambt_19370	1.79	1.57	Hypothetical protein	–
ambt_19395	1.41	1.34	Glutamine synthetase	–
ambt_19400	1.33	1.38	Hypothetical protein	–
ambt_19570	1.05	2.43	Hypothetical protein	R
ambt_19730	3.19	1.17	Hypothetical protein	–
ambt_20010	1.50	1.70	Methyl-accepting chemotaxis protein	NT
ambt_20265	1.77	1.94	Hypothetical protein	–
ambt_20300	2.15	1.41	Hypothetical protein	–
ambt_20315	3.15	3.80	Protein YjbJ	S
ambt_20355	1.61	1.22	Hypothetical protein	J
ambt_20410	1.05	1.02	Purine phosphorylase family protein 1	F
ambt_20665	2.88	1.62	Branched-chain amino acid ABC transporter permease	E
ambt_20670	4.95	3.13	Branched-chain amino acid ABC transporter permease	E
ambt_20675	4.60	4.12	Branched-chain amino acid ABC transporter substrate-binding protein	E
ambt_20680	5.64	5.53	Phosphate-selective porin O and P	–
ambt_20685	1.69	1.07	Two-component hybrid sensor and regulator	T
ambt_20710	3.79	2.28	Transcriptional regulator CadC	–
ambt_20720	3.02	1.68	Hypothetical protein	–
ambt_20755	1.82	1.65	Phage transcriptional regulator, AlpA	K
ambt_20790	1.25	1.02	Hypothetical protein	–
ambt_20795	1.19	1.57	Hypothetical protein	–
ambt_20800	2.05	1.06	Hypothetical protein	–
ambt_20845	1.05	1.90	Hypothetical protein	–
ambt_21175	1.25	2.19	tRNA-dihydrouridine synthase B	J
ambt_21320	1.69	1.79	Hypothetical protein	J
ambt_21325	2.82	1.51	Transposase	L

ambt_21355	1.17	1.44	Osmolarity sensor protein	GEP<br>R
ambt_21440	2.25	1.97	Succinate-semialdehyde dehydrogenase	C
ambt_21475	1.56	1.20	Glucan biosynthesis protein G	P
ambt_21515	1.36	2.07	Hypothetical protein	–
ambt_21640	4.33	2.40	Putative bioH protein	R
ambt_21805	1.59	1.53	Bicyclomycin resistance protein	GEP<br>R
ambt_21810	2.14	1.47	Peptidase S9 prolyl oligopeptidase	E
ambt_21910	1.78	1.74	DedA family protein	S
ambt_21950	1.47	1.28	Phage integrase family site specific recombinase	L
ambt_21955	2.65	2.03	Hypothetical protein	–
ambt_21980	1.29	1.27	Hypothetical protein	–
ambt_22010	1.01	2.26	VrlI like protein	P

Supplementary Table 4. Strain SN2 genes showing a more than two-fold differential expression (> $\log_2$ 2) in response to naphthalene conditions. TF, tidal flat; SW, seawater; P, pyruvate; N, naphthalene.

Gene	Differential gene expression (fold change)		Putative functions	COG category
	$\log_2$ (TF-N/TF-P)	$\log_2$ (SW-N/SW-P)		
ambt_00095	1.42	1.22	16S rRNA methyltransferase B	J
ambt_00550	1.98	2.04	Dipeptidyl peptidase IV	E
ambt_00630	1.35	1.29	Peptide methionine sulfoxide reductase MsrA	O
ambt_00680	2.14	2.04	Carboxylesterase	R
ambt_00745	2.22	3.45	Sulfite reductase subunit alpha	P
ambt_00750	1.83	3.25	Sulfite reductase subunit beta	P
ambt_00755	1.77	1.85	Phosphoadenosine phosphosulfate reductase	EH
ambt_01330	1.71	1.82	Glycosyl hydrolase	–
ambt_01520	1.11	2.25	Hypothetical protein	S
ambt_01595	2.81	1.86	Xenobiotic reductase B	C
ambt_02065	2.82	1.29	Glutathione synthetase	HJ
ambt_02340	5.03	10.85	Membrane-fusion protein	M
ambt_02345	4.46	9.88	RND multidrug efflux transporter MexF	V
ambt_02385	1.81	1.34	D-3-phosphoglycerate dehydrogenase	HE
ambt_02550	3.07	2.52	Putative oxidoreductase	R
ambt_02555	2.77	1.47	Monooxygenase	C
ambt_02795	3.48	1.14	Hypothetical protein	–
ambt_02890	1.10	1.86	Protoheme IX farnesyltransferase	O
ambt_02915	2.78	4.24	Hypothetical protein	–
ambt_02935	2.03	2.22	Hypothetical protein	S
ambt_02940	1.17	1.44	Hypothetical protein	–
ambt_02955	1.46	2.96	Peptidase M1 membrane alanine aminopeptidase	E
ambt_03110	2.26	1.03	Lactoylglutathione lyase	E
ambt_03150	2.62	1.60	Alkyl hydroperoxide reductase/ thiol specific antioxidant/ mal allergen	O
ambt_04470	1.25	1.18	ClpB protein	O
ambt_04575	2.18	1.37	Hypothetical protein	–
ambt_04585	3.91	2.07	Heat shock protein 90	O
ambt_04635	4.21	1.07	GNAT family acetyltransferase	KR
ambt_04665	1.15	1.55	Histidinol-phosphate aminotransferase	E
ambt_04910	1.03	8.19	Putative long-chain-fatty-acid-CoA ligase	I
ambt_04925	1.71	6.15	Hypothetical protein	S
ambt_04930	1.05	6.49	Glycosyl transferase family protein	M
ambt_05545	1.52	2.26	Peptidase M14, carboxypeptidase A	E
ambt_05685	1.98	2.64	TetR family transcriptional regulator	K
ambt_06520	3.03	2.53	Putative DSBA oxidoreductase	Q
ambt_06525	2.92	1.90	Putative xanthine dehydrogenase accessory factor	O
ambt_06765	1.08	5.37	Alkyl hydroperoxide reductase subunit F	O
ambt_06770	2.35	1.17	Hypothetical protein	–
ambt_07225	1.32	2.22	Putative copper resistance (CopC-like) protein	R
ambt_07230	1.19	2.30	Putative copper export protein	P
ambt_07235	1.75	1.64	Hypothetical protein	–
ambt_07340	2.03	1.98	Cation efflux protein	P
ambt_07345	1.38	2.16	Hypothetical protein	–
ambt_07350	1.47	1.77	Co/Zn/Cd efflux system protein	P
ambt_07535	2.15	2.59	Putative glutathione S-transferase	O
ambt_07815	1.80	2.70	Sensor histidine kinase	T
ambt_07820	1.09	2.49	Putative response regulator in two-component regulatory system	KT
ambt_08175	1.90	1.22	Hypothetical protein	–
ambt_08345	4.32	4.12	Hypothetical protein	R
ambt_08995	1.18	2.12	Cysteine synthase A	E
ambt_09025	1.34	1.18	Integrase	L
ambt_09055	5.03	5.68	ACP phosphodiesterase	I
ambt_09205	1.36	2.71	Putative carboxylase	E

ambt_09210	1.34	2.89	Allophanate hydrolase subunit 1	E
ambt_09215	1.61	1.72	LamB/YcsF family protein	R
ambt_09245	1.12	2.42	Hypothetical protein	–
ambt_09940	1.43	3.80	Hypothetical protein	MG
ambt_09945	1.68	1.43	Pyridoxamine 5'-phosphate oxidase-like FMN-binding protein	S
ambt_09950	2.00	1.09	Putative metallo-dependent hydrolase	L
ambt_10925	2.36	1.25	Phage integrase family protein	L
ambt_11330	1.04	1.13	Putative exopolysaccharide biosynthesis protein	M
ambt_11335	1.13	1.25	Polysaccharide export protein	M
ambt_11340	1.03	1.10	Hypothetical protein	–
ambt_11390	1.74	1.40	Hypothetical protein	–
ambt_11435	2.50	1.89	Small heat shock protein	O
ambt_11515	3.28	2.21	Molecular chaperone DnaK	O
ambt_11520	3.70	2.04	Heat shock protein GrpE	O
ambt_11660	1.11	1.12	Bifunctional indole-3-glycerol phosphate synthase/phosphoribosylanthranilate isomerase	E
ambt_11715	1.82	2.25	ABC transporter permease	Q
ambt_11720	1.39	2.01	Putative lipoprotein releasing system ABC transporter ATP-binding protein	Q
ambt_11725	2.36	2.20	Lipase/acylhydrolase, GDSL family protein	E
ambt_11750	1.57	1.97	Zn-dependent enzyme from deacylase/carboxypeptidase superfamily protein	R
ambt_11920	2.94	1.38	Putative multidrug resistance protein	M
ambt_11925	3.36	1.78	Transporter	V
ambt_11935	1.11	1.10	Oxidoreductase, zinc-binding dehydrogenase family protein	CR
ambt_12070	2.95	1.70	Quinolinate synthetase	H
ambt_12095	1.31	1.23	TolA-like protein	M
ambt_12265	1.69	2.12	ABC transporter ATP-binding protein/permease	V
ambt_12270	1.60	2.25	RND family efflux system membrane fusion protein	M
ambt_12275	1.54	1.53	RND family efflux transporter	V
ambt_12280	2.97	2.38	Citrate transporter	P
ambt_12350	1.71	1.00	Hypothetical protein	–
ambt_12365	2.46	3.18	Putative ATPase family protein	R
ambt_12370	2.51	2.75	Hypothetical protein	R
ambt_12375	2.54	2.87	Hypothetical protein	–
ambt_12380	3.04	3.39	Von Willebrand factor, type A	R
ambt_12385	2.93	2.54	Hypothetical protein	R
ambt_12390	2.55	2.22	BatD protein	–
ambt_12395	1.33	1.46	Hypothetical protein	S
ambt_12485	1.47	1.09	Hypothetical protein	–
ambt_12550	1.52	2.98	Acylaminoacyl-peptidase	E
ambt_12725	2.17	1.42	Hypothetical protein	S
ambt_12730	2.30	1.62	Glutamate cysteine ligase	H
ambt_12735	1.37	1.07	Putative metallopeptidase	R
ambt_12855	1.90	1.64	Hypothetical protein	–
ambt_13100	1.00	1.93	Hypothetical protein	S
ambt_13905	1.80	1.09	Thiol:disulfide interchange protein dsbE	OC
ambt_13915	1.45	1.06	Cytochrome c-type biogenesis protein CcmE	O
ambt_13920	1.65	1.29	Heme exporter protein CcmD	U
ambt_13995	2.67	3.14	Alpha/beta hydrolase fold protein	R
ambt_14055	2.24	3.10	Hypothetical protein	–
ambt_14095	2.05	2.36	Hypothetical protein	–
ambt_14455	1.99	1.25	Cell volume regulation protein CvrA	P
ambt_14460	5.26	2.41	Acriflavin resistance protein	U
ambt_14465	5.24	1.47	Hypothetical protein	–
ambt_14530	1.84	1.99	Protease DO	O
ambt_14540	2.91	2.35	UDP-N-acetylglucosamine 1-carboxyvinyltransferase	M
ambt_14545	2.90	2.29	BolA-like protein	K
ambt_14550	3.03	3.24	STAS domain-containing protein	R
ambt_14555	2.81	3.18	Toluene tolerance family protein	Q
ambt_14560	2.89	3.54	Mce family protein	Q
ambt_14565	2.97	3.44	ABC transporter membrane protein	Q

ambt_14570	3.22	2.81	Putative ABC transporter ATP-binding protein	Q
ambt_14710	1.81	1.05	30S Ribosomal protein S9	J
ambt_14715	2.45	1.18	50S Ribosomal protein L13	J
ambt_14770	2.07	1.88	Thiol:disulfide interchange protein DsbC	O
ambt_14870	6.32	2.32	Hypothetical protein	–
ambt_15040	1.28	3.18	HlyD family secretion protein	M
ambt_15115	2.08	1.66	Thiol:disulfide interchange protein DsbA	OC
ambt_15120	1.45	1.71	Putative hydrolase	R
ambt_15205	1.48	1.99	PA-phosphatase-like phosphoesterase	I
ambt_15315	2.63	1.89	Outer membrane channel protein	MU
ambt_15815	2.48	1.25	Thioredoxin domain-containing protein	O
ambt_15870	1.52	1.02	Thioredoxin	OC
ambt_16085	1.34	1.41	Ornithine carbamoyltransferase	E
ambt_16090	1.26	1.27	Argininosuccinate synthase	E
ambt_16095	1.47	1.27	Argininosuccinate lyase	E
ambt_16100	1.77	1.09	N-acetylglutamate synthase	E
ambt_16130	3.27	2.84	Putative glutathione S-transferase	O
ambt_16450	3.22	2.64	Formyltetrahydrofolate deformylase	–
ambt_16510	1.47	1.30	30S Ribosomal protein S3	J
ambt_16515	2.21	1.63	50S Ribosomal protein L22	J
ambt_16520	2.06	1.54	30S Ribosomal protein S19	J
ambt_16525	1.62	1.65	50S Ribosomal protein L2	J
ambt_16530	2.15	1.80	50S Ribosomal protein L23	J
ambt_16535	2.92	1.52	50S Ribosomal protein L4	J
ambt_16540	1.71	1.56	50S Ribosomal protein L3	J
ambt_16545	2.36	1.54	30S Ribosomal protein S10	J
ambt_16700	3.34	2.05	Hypothetical protein	–
ambt_16705	2.48	1.35	Heat shock protein HtpX	O
ambt_17425	2.28	1.79	Lipoprotein	S
ambt_17500	1.76	2.14	Serine acetyltransferase	E
ambt_17980	5.24	5.53	Fumarylacetoacetate (FAA) hydrolase	Q
ambt_17985	5.37	4.93	Cupin	Q
ambt_17990	4.56	5.23	Maleylacetoacetate isomerase	O
ambt_18005	4.40	8.18	Rieske (2Fe-2S) domain-containing protein	PR
ambt_18010	4.14	8.14	Aromatic-ring-hydroxylating dioxygenase subunit beta	Q
ambt_18015	3.79	7.97	Rieske (2Fe-2S) domain-containing protein	PR
ambt_18020	2.35	5.81	Hypothetical protein	–
ambt_18025	2.95	5.16	Hypothetical protein	–
ambt_18030	3.07	5.26	Hypothetical protein	–
ambt_18035	3.39	5.72	Ferredoxin oxidoreductase protein	HC
ambt_18040	3.50	5.53	Hypothetical protein	–
ambt_18180	2.12	1.02	Hypothetical protein	R
ambt_18185	2.58	1.44	Acriflavine resistance protein B	V
ambt_18190	2.58	1.91	Acriflavine resistance protein a	M
ambt_18270	1.18	1.01	Putative ABC transporter permease	P
ambt_18360	1.14	2.25	NCS1 nucleoside transporter	FH
ambt_18370	2.70	2.05	Methylmalonate-semialdehyde dehydrogenase	C
ambt_18375	2.19	2.65	Beta alanine--pyruvate transaminase	H
ambt_18415	1.02	1.47	NADPH-dependent F420 reductase	R
ambt_18425	1.15	1.65	TonB-dependent receptor	P
ambt_19200	4.89	1.86	Helix-turn-helix domain-containing protein	K
ambt_19205	5.08	1.97	Quinol oxidase subunit I	C
ambt_19210	4.34	1.10	Quinol oxidase subunit II	C
ambt_19260	2.63	1.93	3-Methyl-adenine DNA glycosylase	L
ambt_19265	2.01	2.23	Oligopeptidase A	E
ambt_19340	3.53	2.29	ATP-dependent protease ATP-binding subunit HslU	O
ambt_19345	3.19	2.95	ATP-dependent protease subunit HslV	O
ambt_19470	2.66	1.10	Aminopeptidase B	E
ambt_19475	2.39	2.31	Hypothetical protein	E
ambt_19525	2.22	1.99	Putative methyltransferase SAM dependent	H
ambt_19530	2.12	1.91	Inner membrane transport permease	V
ambt_19535	3.02	1.92	ABC transporter ATP-binding protein	V

ambt_19575	1.51	1.52	Permease	R
ambt_19655	1.53	1.33	50S Ribosomal protein L11	J
ambt_19720	1.60	2.66	Aldo/keto reductase family oxidoreductase	R
ambt_19725	1.24	2.81	Glyoxylase I family protein	E
ambt_19975	3.04	1.09	50S Ribosomal protein L9	J
ambt_19980	2.30	1.28	30S Ribosomal protein S18	J
ambt_20315	2.19	1.53	Protein YjbJ	S
ambt_20375	1.46	5.11	CBS domain-containing protein	R
ambt_20385	4.24	4.04	Putative cytochrome b561	C
ambt_20390	4.41	4.68	YceI protein	S
ambt_20415	1.05	3.59	LysR family transcriptional regulator	K
ambt_20420	2.49	1.64	Long-chain fatty acid transport protein	I
ambt_20430	2.10	1.48	Putative NADP-dependent oxidoreductase	R
ambt_20460	1.12	2.13	Flavin dependent oxidoreductase	C
ambt_20540	1.40	1.80	DeoR family transcriptional regulator	KG
ambt_21005	2.04	1.14	Integrase family protein	L
ambt_21010	3.95	2.36	Chaperonin GroEL	O
ambt_21015	4.94	2.47	Co-chaperonin GroES	O
ambt_21020	2.43	3.24	FxsA protein	R
ambt_21120	2.28	6.04	Cytochrome b561	C
ambt_21125	2.94	7.39	Hypothetical protein	–
ambt_21130	2.91	7.88	Aldo/keto reductase	C
ambt_21135	2.63	7.63	Short-chain dehydrogenase/reductase SDR	IQR
ambt_21140	3.08	7.40	Hypothetical protein	S
ambt_21650	1.53	1.72	Peptidase M14, carboxypeptidase A	L
ambt_21655	2.70	1.34	Fe/S biogenesis protein NfuA	S
ambt_21660	2.22	1.14	Hypothetical protein	–
ambt_21805	1.62	1.68	Bicyclomycin resistance protein	GEPR
ambt_21860	1.61	2.34	Putative Zn-dependent aminopeptidase	E
ambt_21865	1.86	2.03	Hypothetical protein	–

Supplementary Table 5. Strain SN2 genes showing a more than two-fold differential expression (> $\log_2$ 2) in response to pyruvate conditions. TF, tidal flat; SW, seawater; P, pyruvate; N, naphthalene.

Gene	Differential gene expression (fold change)		Putative functions	COG category
	$\log_2$ (TF-P/TF-N)	$\log_2$ (SW-P/SW-N)		
ambt_00080	2.35	1.12	TraT complement resistance	–
ambt_00255	2.65	1.15	Methyl-accepting chemotaxis sensory transducer	–
ambt_00350	8.77	2.63	Hypothetical protein	R
ambt_00525	1.10	1.48	Putative metallo-beta-lactamase	R
ambt_00530	1.46	1.54	Putative metal-dependent amidohydrolase	R
ambt_00535	2.01	1.74	Threonine dehydratase	E
ambt_00570	1.69	1.95	Hypothetical protein	–
ambt_00595	3.09	4.22	Rhs family protein	U
ambt_00660	1.81	1.37	Hypothetical protein	–
ambt_00810	3.19	1.22	Hypothetical protein	–
ambt_00815	2.74	2.78	RNA-binding protein	R
ambt_00915	4.37	2.16	GntR family transcriptional regulator	K
ambt_00980	1.67	1.72	Hypothetical protein	–
ambt_00985	2.42	1.59	Hypothetical protein	NT
ambt_00990	1.32	1.60	Hypothetical protein	T
ambt_00995	1.42	1.46	Hypothetical protein	U
ambt_01000	2.52	1.40	Hypothetical protein	L
ambt_01005	2.02	1.34	Serine/threonine protein phosphatase, putative	T
ambt_01010	1.86	1.93	Forkhead-associated protein	O
ambt_01015	1.19	2.06	Hypothetical protein	–
ambt_01080	2.42	1.02	GntR family transcriptional regulator	K
ambt_01095	1.69	2.68	Two component LuxR family transcriptional regulator	TK
ambt_01100	6.83	5.95	Aldehyde dehydrogenase family protein	C
ambt_01105	4.00	2.75	FMN-binding domain-containing protein	K
ambt_01110	2.93	2.74	Hypothetical protein	S
ambt_01115	1.88	1.91	PAS/PAC sensor-containing hybrid histidine kinase	T
ambt_01120	5.52	3.24	Two component LuxR family transcriptional regulator	TK
ambt_01130	1.35	1.59	Hypothetical protein	–
ambt_01150	2.28	3.19	Pyrroloquinoline quinone biosynthesis protein PqqE	R
ambt_01155	3.27	4.45	Pyrroloquinoline quinone biosynthesis protein PqqD	–
ambt_01160	3.61	3.70	Pyrroloquinoline quinone biosynthesis protein PqqC	H
ambt_01165	4.96	4.35	Pyrroloquinoline quinone biosynthesis protein PqqB	R
ambt_01185	1.75	2.89	Hypothetical protein	S
ambt_01190	1.69	2.56	Hypothetical protein	–
ambt_01195	1.87	2.14	Hypothetical protein	ET
ambt_01200	3.66	2.66	Methanol/ethanol family PQQ-dependent dehydrogenase	G
ambt_01205	4.91	5.94	Cytochrome c-550	C
ambt_01210	4.36	1.25	Hypothetical protein	R
ambt_01280	1.54	1.75	Hypothetical protein	–
ambt_01440	3.96	1.23	Hypothetical protein	–
ambt_01670	1.50	1.61	Hypothetical protein	–
ambt_01970	3.46	2.11	Hypothetical protein	–
ambt_02045	1.18	1.21	Twitching motility protein PilU (type IV pili)	NU
ambt_02075	1.29	1.99	Hypothetical protein	–
ambt_02110	3.64	3.68	ADP-ribosylglycohydrolase family protein	S
ambt_02115	3.78	3.54	Hypothetical protein	T
ambt_02275	1.80	2.76	Type 4 prepilin-like proteins leader peptide processing enzyme	NOU
ambt_02280	2.89	4.10	Type IV pilus biogenesis protein PilC	NU
ambt_02285	4.36	4.40	Type IV prepilin TapA	NU
ambt_02305	1.48	1.05	Regulatory protein AmpE	V
ambt_02365	1.83	2.39	Fe-S protein-like protein EB3	R
ambt_02465	2.07	1.33	Glycine cleavage system protein H	E
ambt_02695	1.40	1.07	Hypothetical protein	–
ambt_02750	1.53	1.04	Late embryogenesis abundant protein	–
ambt_02755	1.51	1.09	Hypothetical protein	–
ambt_03020	3.77	1.02	Hypothetical protein	–

ambt_03035	3.74	2.52	Hypothetical protein	M
ambt_03040	4.58	3.03	Hypothetical protein	–
ambt_03045	3.50	3.12	Hypothetical protein	–
ambt_03050	2.02	2.84	Hypothetical protein	–
ambt_03490	2.02	1.11	Putative integral membrane protein	R
ambt_03500	1.14	1.06	Hypothetical protein	–
ambt_03680	2.72	1.29	Hypothetical protein	H
ambt_03685	2.76	1.29	Hypothetical protein	J
ambt_03875	1.03	2.06	Hypothetical protein	–
ambt_04105	2.86	2.44	Bile acid:sodium symporter	R
ambt_04110	2.52	1.46	Short chain dehydrogenase	IQR
ambt_04115	2.45	1.78	Putative aminoglycoside phosphotransferase	R
ambt_04160	3.51	2.57	Hypothetical protein	–
ambt_04165	3.11	3.14	Pilin like competence factor	NU
ambt_04170	3.40	3.72	Hypothetical protein	–
ambt_04175	2.93	2.78	Tfp pilus assembly protein tip-associated adhesin PilY1-like protein	NU
ambt_04180	1.91	2.93	General secretion pathway protein H	NU
ambt_04210	2.13	2.40	Putative type IV pilus biogenesis protein	NU
ambt_04485	3.54	4.23	Hypothetical protein	–
ambt_04510	1.83	1.11	Hypothetical protein	–
ambt_04530	3.18	4.05	Hypothetical protein	–
ambt_04535	1.51	2.87	Hypothetical protein	S
ambt_04540	1.89	2.18	Hypothetical protein	S
ambt_04560	1.30	5.04	Vanadium-dependent bromoperoxidase 2	–
ambt_04610	5.39	1.86	TonB-dependent receptor	P
ambt_04685	1.48	1.37	Hypothetical protein	–
ambt_04720	4.65	1.17	Hypothetical protein	S
ambt_04820	3.58	2.32	Hypothetical protein	R
ambt_04970	1.19	2.15	Putative Oxidoreductase	C
ambt_05040	3.25	1.80	Class II aldolase/adducin-like protein	G
ambt_05090	1.33	1.34	Hypothetical protein	–
ambt_05145	2.50	4.40	Hypothetical protein	–
ambt_05150	1.10	1.22	Hypothetical protein	L
ambt_05485	4.26	1.05	Hypothetical protein	–
ambt_05530	2.27	1.26	Exopolyphosphatase-like protein	R
ambt_05615	1.05	1.26	Putative diguanylate cyclase/phosphodiesterase	T
ambt_06045	2.76	2.41	AMP-binding protein	I
ambt_06080	1.04	2.31	Hypothetical protein	E
ambt_06135	1.71	1.07	Hypothetical protein	S
ambt_06560	2.69	3.36	Hypothetical protein	–
ambt_06565	6.82	1.18	Short-chain dehydrogenase/reductase SDR	IQR
ambt_06570	7.10	1.92	Haloalkane dehalogenase	R
ambt_06575	6.92	1.30	3-Hydroxyacyl-CoA dehydrogenase	I
ambt_06590	4.31	3.53	Alpha/beta hydrolase fold protein	R
ambt_06595	2.94	5.11	Hypothetical protein	–
ambt_06620	3.09	4.84	Hypothetical protein	–
ambt_06630	2.56	1.46	Acyl dehydratase	I
ambt_06660	8.44	3.14	TonB-dependent receptor	P
ambt_06750	1.28	3.33	Putative extracellular nuclease	R
ambt_07010	1.10	2.90	Hypothetical protein	–
ambt_07050	2.38	1.32	TonB-dependent receptor	P
ambt_07115	2.99	1.07	Hypothetical protein	–
ambt_07420	2.87	1.09	Hypothetical protein	–
ambt_07600	3.96	1.05	TonB-dependent receptor	P
ambt_07685	3.21	2.89	Hypothetical protein	R
ambt_07690	5.57	3.58	Penicillin acylase-like protein	R
ambt_07825	2.44	1.33	6-phosphofructokinase	G
ambt_07830	1.97	2.04	Hypothetical protein	–
ambt_07835	2.87	1.80	Response regulator with TPR repeat	T
ambt_07840	1.36	1.12	Putative fumarate reductase flavoprotein subunit	–
ambt_07910	8.48	1.02	Putative succinyl-CoA transferase subunit alpha	I
ambt_07915	2.56	1.34	Putative transcriptional regulator	K

ambt_07960	2.71	1.53	Peptidase S8/S53 subtilisin kexin sedolisin	O
ambt_08050	4.18	3.39	Hypothetical protein	–
ambt_08055	3.68	2.78	Hypothetical protein	–
ambt_08060	3.28	2.40	BNR repeat-containing protein	R
ambt_08065	1.69	1.60	Hypothetical protein	R
ambt_08105	1.96	1.30	3-Hydroxydecanoyl-(acyl carrier protein) dehydratase	I
ambt_08165	1.10	2.20	Hypothetical protein	–
ambt_08370	2.77	1.56	MarR family transcriptional regulator	K
ambt_08390	1.07	1.03	Response regulator receiver domain-containing protein	TK
ambt_08395	1.88	1.18	Interphotoreceptor retinoid-binding protein	M
ambt_08645	1.57	1.17	Hypothetical protein	–
ambt_08825	1.95	1.57	Hypothetical protein	–
ambt_09085	2.62	1.97	Short chain dehydrogenase	IQR
ambt_09090	3.69	2.83	Short-chain alcohol dehydrogenase-like protein	IQR
ambt_09095	4.41	3.44	Acyl-CoA dehydrogenase-like protein	I
ambt_09100	1.67	2.19	AraC family transcriptional regulator	K
ambt_09125	1.31	1.44	Isoamylase protein-like protein	G
ambt_09165	3.28	1.67	Methyl-accepting chemotaxis protein	NT
ambt_09185	1.18	1.58	Gluconate transporter	GE
ambt_09250	8.34	4.57	Putative cytochrome c oxidase subunit I	–
ambt_09405	2.43	1.74	Restriction endonuclease	V
ambt_09410	1.96	1.72	Mechanosensitive ion channel MscS	M
ambt_09440	1.88	1.66	Hypothetical protein	–
ambt_09505	4.26	1.69	NAD-dependent epimerase/dehydratase:Short-chain dehydrogenase/reductase SDR	IQR
ambt_09600	4.70	1.61	DoxX protein	S
ambt_09665	2.92	1.49	Stress response protein CspD	K
ambt_09705	4.72	1.67	Hypothetical protein	KR
ambt_09805	1.56	1.31	Hypothetical protein	–
ambt_09825	1.31	1.01	Short-chain dehydrogenase/reductase SDR	IQR
ambt_09850	1.96	1.05	Integral membrane protein TerC	–
ambt_10015	2.58	2.06	Maf protein	D
ambt_10130	1.25	3.21	Invasin domain-containing protein	–
ambt_10190	3.40	1.08	Type IV pilus assembly PilZ	–
ambt_10220	1.50	2.47	Acid phosphatase	R
ambt_10230	6.62	1.98	CBS domain-containing protein	R
ambt_10270	4.70	1.52	Acetyl-CoA acetyltransferase with thiolase domain	I
ambt_10325	2.23	1.75	Hypothetical protein	–
ambt_10345	6.19	1.73	Major facilitator family transporter	GEPR
ambt_10485	2.66	1.17	Hypothetical protein	–
ambt_10565	1.56	1.87	Transposase	–
ambt_10875	2.60	4.30	TPR repeat- and GGDEF domain-containing signaling protein	T
ambt_11000	5.90	1.71	Homogentisate 1,2-dioxygenase	Q
ambt_11060	1.14	2.35	Sensory box/GGDEF domain/EAL domain-containing protein	T
ambt_11080	4.86	3.96	MoxR protein	R
ambt_11085	3.28	3.62	Hypothetical protein	R
ambt_11090	2.83	2.53	Transglutaminase family protein	E
ambt_11440	1.17	1.29	Hypothetical protein	–
ambt_11525	1.87	1.10	Hypothetical protein	ER
ambt_11530	6.87	3.52	LuxR family response regulator	TK
ambt_11940	3.11	1.31	Zinc-binding alcohol dehydrogenase	CR
ambt_11980	1.45	3.78	2-dehydropantoate 2-reductase	H
ambt_11985	2.04	4.94	Phytanoyl-CoA dioxygenase	Q
ambt_12260	1.37	1.37	O-Acetylhomoserine/O-acetylserine sulfhydrylase	E
ambt_12285	3.27	2.59	Hypothetical protein	–
ambt_12305	3.04	1.20	Hypothetical protein	–
ambt_12400	1.55	1.22	RNA polymerase sigma factor	K
ambt_12410	1.24	1.86	Response regulator receiver modulated diguanylate cyclase	T
ambt_12715	3.35	1.43	Hypothetical protein	–
ambt_12965	1.97	2.40	TonB-dependent receptor	P
ambt_13005	1.90	2.09	Transcriptional regulatory protein tyrR	KE

ambt_13010	6.58	1.29	Phenylalanine 4-monooxygenase	E
ambt_13115	1.13	2.96	TonB-dependent receptor	P
ambt_13315	3.19	2.09	Flagellin-like protein	N
ambt_13415	1.22	1.04	Hypothetical protein	–
ambt_13420	1.94	1.16	Flagellar protein FliS	NUO
ambt_13425	2.42	1.05	Flagellar hook-associated 2-like protein	N
ambt_13430	2.99	1.03	Flagellar protein FlaG protein	N
ambt_13435	4.41	1.89	Flagellin-like protein	N
ambt_13450	2.20	1.91	Flagellin-like protein	N
ambt_13530	1.81	1.13	Negative regulator of flagellin synthesis FlgM	KNU
ambt_13535	1.33	1.03	Putative flagellar protein FlgN	–
ambt_13550	1.36	1.56	Hypothetical protein	S
ambt_13555	1.48	1.39	Diguanylate cyclase/phosphodiesterase	T
ambt_13560	2.91	1.25	Putative LuxR-family transcriptional regulator	TK
ambt_13570	2.62	1.22	ABC transporter ATP-binding protein/permease	V
ambt_13580	5.90	2.86	Hypothetical protein	–
ambt_13755	1.98	1.42	Hypothetical protein	–
ambt_13765	2.43	1.14	DNA-directed DNA polymerase	L
ambt_13770	2.45	2.33	Hypothetical protein	S
ambt_13775	3.06	1.90	Putative GNAT family acetyltransferase	KR
ambt_14070	1.97	1.00	Sensory box/GGDEF protein	T
ambt_14100	2.61	1.12	Hypothetical protein	–
ambt_14135	1.10	2.18	Transposase	–
ambt_14180	1.99	1.92	Hypothetical protein	–
ambt_14260	1.67	1.92	Pas/Pac sensor containing methyl-accepting chemotaxis sensory transducer	T
ambt_14290	5.93	1.13	Hypothetical protein	–
ambt_14310	1.37	2.18	Putative hemolysin III	R
ambt_14340	1.17	1.33	Universal stress protein UspA	T
ambt_14410	4.97	2.02	Hypothetical protein	S
ambt_14415	4.43	1.06	Hypothetical protein	–
ambt_14815	1.50	2.88	Methyl-accepting chemotaxis protein	NT
ambt_14855	4.19	2.02	Long-chain-fatty-acid--CoA ligase	IQ
ambt_15020	1.47	1.90	Methyl-accepting chemotaxis protein	NT
ambt_15025	4.33	2.41	Topoisomerase IV subunit B	–
ambt_15030	4.45	2.04	Phosphate ABC transporter substrate-binding protein	–
ambt_15365	1.40	2.07	Hypothetical protein	–
ambt_15465	5.87	1.92	Putative chemotaxis sensory protein	NT
ambt_15510	1.84	1.38	Putative lipoprotein NlpD	M
ambt_15515	3.14	1.86	Sigma S (sigma 38) factor of RNA polymerase, major sigma factor during stationary phase	K
ambt_16000	1.84	1.02	Hypothetical protein	–
ambt_16120	1.78	1.50	Hypothetical protein	ER
ambt_16150	1.63	2.08	Putative type IV pilus biogenesis protein PilM	NU
ambt_16155	1.94	2.39	Type IV pilus biogenesis protein PilN	NU
ambt_16160	2.38	2.29	Type IV pilus biogenesis protein PilO	NU
ambt_16165	1.49	2.36	Putative type IV pilus biogenesis protein PilP	NU
ambt_16170	1.44	1.84	Putative type IV pilus biogenesis protein PilQ	U
ambt_16570	2.42	1.31	Iron-containing alcohol dehydrogenase	C
ambt_16600	1.28	1.09	Putative multi-domain-containing protein	–
ambt_16605	1.97	1.42	Twin-arginine translocation pathway signal protein	–
ambt_16610	1.77	1.68	Oxidoreductase	E
ambt_16620	1.17	2.11	Putative nucleoside permease	GEP R
ambt_16625	2.60	1.28	Xylose isomerase-like protein	G
ambt_16635	1.75	1.36	LacI family transcriptional repressor	K
ambt_16640	1.49	1.91	Twin-arginine translocation pathway signal protein	G
ambt_16715	2.20	3.08	Hypothetical protein	–
ambt_16720	2.41	1.03	Chemotaxis-specific methylesterase	NT
ambt_16725	1.81	1.01	Protein CheD	NT
ambt_16735	3.76	1.10	Pas/Pac sensor containing methyl-accepting chemotaxis sensory transducer	T

ambt_17175	2.01	2.07	Prepilin-type cleavage/methylation protein	–
ambt_17180	3.04	2.24	Methylation	–
ambt_17305	3.06	1.56	Platelet-activating factor acetylhydrolase, plasma/intracellular isoform II	R
ambt_17325	3.69	1.05	Diguanylate phosphodiesterase	T
ambt_17330	3.30	1.25	Intracellular signaling protein	T
ambt_17360	1.09	2.24	Putative sodium/hexose cotransport protein	R
ambt_17365	2.96	2.35	Hypothetical protein	–
ambt_17600	2.10	1.39	Hypothetical protein	–
ambt_17680	1.47	1.42	Hypothetical protein	–
ambt_17695	2.60	2.06	Hypothetical protein	U
ambt_17775	1.69	1.50	Hypothetical protein	–
ambt_17780	1.52	1.06	Hypothetical protein	–
ambt_17840	1.64	1.14	Hypothetical protein	–
ambt_17870	1.52	1.17	Hypothetical protein	–
ambt_17875	1.78	1.24	Aromatic hydrocarbon degradation membrane protein	–
ambt_17885	1.86	1.56	2,3-dihydroxy-2,3-dihydrophenylpropionate dehydrogenase	IQR
ambt_17890	2.19	1.91	Aromatic-ring-hydroxylating dioxygenase subunit beta	Q
ambt_17895	2.47	2.26	Ring hydroxylating dioxygenase subunit alpha	PR
ambt_17900	2.00	2.31	Protocatechuate 4,5-dioxygenase subunit beta	–
ambt_17905	2.18	2.20	Hypothetical protein	–
ambt_17910	2.10	2.38	Dihydrodipicolinate synthetase	EM
ambt_17915	2.46	2.79	Aldehyde dehydrogenase	C
ambt_17920	1.80	2.93	Putative ferredoxin	PR
ambt_17925	1.39	3.11	Fumarylacetoacetate (FAA) hydrolase	Q
ambt_17930	1.31	2.90	Cupin	Q
ambt_17950	1.89	1.08	2-Hydroxyhepta-2,4-diene-1,7-dioate isomerase	Q
ambt_18540	10.49	1.33	Methylmalonate-semialdehyde dehydrogenase	C
ambt_18545	2.93	1.12	Transcriptional regulatory protein	K
ambt_18580	1.23	1.00	Hypothetical protein	–
ambt_18855	2.74	2.70	Hypothetical protein	Q
ambt_18910	2.28	1.48	Hypothetical protein	–
ambt_18985	2.35	1.57	Transposase	–
ambt_19000	2.18	2.03	Hypothetical protein	–
ambt_19135	6.06	5.71	Cold-active serine alkaline protease	O
ambt_19180	1.04	1.12	Hypothetical protein	–
ambt_19215	3.42	1.72	Methyl-accepting chemotaxis sensory transducer	NT
ambt_19325	3.56	1.74	Putative LuxR family transcriptional regulator	TK
ambt_19330	3.91	1.76	Hypothetical protein	–
ambt_19605	4.98	1.35	Hydroxymethylglutaryl-coenzyme A reductase	I
ambt_19770	4.93	1.55	Hypothetical protein	–
ambt_19775	2.51	1.57	Sugar kinase, ribokinase family protein	G
ambt_20075	3.01	1.48	Hypothetical protein	G
ambt_20130	1.48	2.12	Hypothetical protein	–
ambt_20135	1.58	1.06	Hypothetical protein	NU
ambt_20365	1.89	1.30	Hypothetical protein	O
ambt_20945	1.19	1.27	Hypothetical protein	–
ambt_21200	1.33	1.03	Hypothetical protein	–
ambt_21300	3.43	1.04	Transposase, IS4 family protein	–
ambt_21730	6.86	2.94	Cytochrome c oxidase subunit III	C
ambt_21735	10.19	3.25	Cytochrome C oxidase assembly protein	O
ambt_21740	8.38	3.86	Cytochrome c oxidase subunit I	C
ambt_21745	9.70	4.90	Cytochrome c oxidase subunit II	C
ambt_21755	1.58	3.96	Putative TonB-dependent receptor	P
ambt_21885	4.15	2.40	Hypothetical protein	I
ambt_21920	1.46	1.32	Hypothetical protein	–
ambt_22020	1.69	1.12	Hypothetical protein	K
ambt_22060	4.69	1.11	Hypothetical protein	–
ambt_22065	1.20	1.25	Hypothetical protein	S
